# Vitamin D Receptor Polymorphisms and Rheumatoid Arthritis Risk: A Systematic Review and Meta-Analysis Evaluating the Moderating Effects of Ethnicity, Bone Erosion, and Classification Criteria

**DOI:** 10.5152/ArchRheumatol.2026.25079

**Published:** 2026-04-27

**Authors:** WeiLin Liu, Su Xi

**Affiliations:** 1 Department of Allergy, Qingdao Municipal Hospital (East Hospital), Qingdao, China; 2 Department of General Medicine, Qingdao Municipal Hospital, Qingdao, China

**Keywords:** ApaI, BsmI, fokI, rheumatoid arthritis, vitamin D receptor

## Abstract

**Background/Aims:**

Previous studies examining the association between vitamin D receptor (VDR) polymorphisms (ApaI, BsmI, TaqI, FokI) and rheumatoid arthritis (RA) have reported inconsistent findings. This meta-analysis aimed to evaluate whether clinical and population-level factors moderate this association.

**Materials and Methods:**

A systematic review of PubMed, Web of Science, Scopus, Cochrane Library, ClinicalTrials.gov, and Google Scholar was conducted up to July 19, 2024. Observational studies comparing VDR polymorphisms between RA patients and healthy controls were included. Studies without a control group, with fewer than 20 cases, or lacking relevant outcome data were excluded. Methodological quality was assessed using the Newcastle–Ottawa Scale. Random-effects meta-analyses, subgroup analyses, and meta-regression were performed.

**Results:**

Twenty-five case-control studies (3711 RA patients and 3884 controls) were included. No significant overall association was observed between VDR polymorphisms and RA across genetic models. However, subgroup analyses demonstrated ethnic variability, with reduced RA likelihood observed among South Asians for the ApaI dominant model (OR = 0.26, 95% CI: 0.14-0.49) and BsmI polymorphism, while African populations showed increased likelihood for BsmI under the recessive model (OR = 1.77, 95% CI: 1.13-2.78). Meta-regression identified ethnicity, classification criteria, and bone erosion as significant moderators; bone erosion was associated with increased RA likelihood for the FokI dominant model (OR = 2.21, 95% CI: 1.24-3.97).

**Conclusion:**

Although no consistent overall association was identified, the relationship between VDR polymorphisms and RA appears to be modified by ethnicity, classification criteria, and disease severity. These findings underscore the importance of accounting for clinical and population heterogeneity in genetic association studies of RA.

**Main Points**

- Ethnicity significantly modified the association between vitamin D receptor polymorphisms and rheumatoid arthritis, with South Asians showing reduced likelihood and Africans demonstrating an opposite pattern.
- Classification criteria (1987 ACR vs. 2010 ACR/EULAR) influenced the observed associations, underscoring the importance of standardized rheumatoid arthritis (RA) definitions.
- Bone erosion was a key clinical moderator, strengthening the association between FokI polymorphism and RA likelihood.
- Subgroup and meta-regression analyses provided a comprehensive understanding of genetic and contextual factors shaping RA susceptibility.
- These findings highlight the need for precision medicine approaches that integrate both genetic variants and clinical moderators in RA.

## Introduction

Rheumatoid arthritis (RA) is a chronic autoimmune disorder characterized by persistent synovial inflammation, leading to joint destruction, disability, and systemic complications.^1^ The etiology of RA is multifactorial, with both genetic and environmental factors playing critical roles in disease onset and progression.^2^ Among the various genetic determinants implicated in RA, the vitamin D receptor (VDR) gene has garnered significant attention due to its potential role in immune modulation and bone metabolism.^3^

Vitamin D exerts its biological effects through the VDR, a nuclear receptor that regulates the expression of numerous genes involved in immune responses and calcium homeostasis.^4^ The VDR gene, located on chromosome 12q13.11, harbors several polymorphisms that have been studied for their association with various autoimmune diseases, including RA.^5^ The most commonly investigated VDR polymorphisms include ApaI (rs7975232), BsmI (rs1544410), TaqI (rs731236), and FokI (rs2228570).^5^ These polymorphisms have been hypothesized to influence VDR gene expression, receptor function, or the downstream effects of vitamin D, potentially contributing to the pathogenesis of RA.^6^^-^^8^

The relationship between VDR polymorphisms and RA risk has been explored in numerous studies across different populations.^3^^,^^8^^-^^12^ However, the findings have been inconsistent, with some studies reporting a significant association between specific VDR polymorphisms and RA, while others have found no such relationship. These discrepancies may be attributed to differences in study design, sample size, population characteristics, and the genetic background of the participants. Moreover, the potential interaction between VDR polymorphisms and other risk factors, such as vitamin D levels, bone mineral density (BMD), and RA disease activity, has been largely unexplored in the literature.

Previous systematic reviews and meta-analyses have examined the association between VDR polymorphisms and rheumatoid arthritis; however, their findings remain inconsistent and limited by substantial heterogeneity across studies. Importantly, most prior reviews focused primarily on direct genetic associations and did not comprehensively investigate potential clinical and population-level moderators that may explain variability in results.^5^ Furthermore, inconsistencies in classification criteria, absence of analyses accounting for disease severity, and limited evaluation of contextual factors such as ethnicity and bone erosion have restricted interpretation of existing evidence.

Therefore, this systematic review and meta-analysis aimed to evaluate the association between VDR polymorphisms (ApaI, BsmI, TaqI, and FokI) and rheumatoid arthritis risk in adult patients compared with healthy controls. In addition, it was aimed to determine whether clinical and population-level factors, including ethnicity, classification criteria, and bone erosion, modify this association. The primary outcome was the pooled odds ratio for rheumatoid arthritis occurrence across different genetic models, while secondary analyses included subgroup and meta-regression analyses to identify potential moderators of the association.

## Materials and Methods

### Study Design and Data Sources

This study was designed as a systematic review and meta-analysis of studies reporting the association between VDR genes and rheumatoid arthritis occurrence. The study followed the Preferred Reporting Items for Systematic Reviews and Meta-Analyses (PRISMA) guidelines.^13^ This systematic review and meta-analysis was not prospectively registered in PROSPERO. Protocol registration was not pursued due to the current PROSPERO screening process, which may decline protocols considered substantially similar to existing registrations. Given the presence of prior systematic reviews investigating VDR polymorphisms in RA, potential overlap was anticipated despite the moderator-focused analytical framework. Retrospective registration was also not performed, as PROSPERO does not permit registration after the screening phase has commenced. Ethical approval and informed consent were not required for this study, as it is a systematic review and meta-analysis based solely on data from previously published studies and does not involve direct interaction with human participants or access to identifiable individual-level data.

A systematic literature search was carried out across various databases, including PubMed, Web of Science, Scopus, Cochrane Library, and clinicaltrials.gov from inception until July 19, 2024. The first 200 records of Google Scholar were also retrieved, as per recent guidelines to maintain relevance to the research question.^14^ Additional manual searches were performed to identify relevant studies not captured in the database search. This was done through: (1) screening the citations of finally included studies, (2) using the “similar articles” function on PubMed, and (3) manually reviewing results on Google Software.^15^ The search query consisted of a set of keywords and Medical Subject Headings (MeSH) terms related to rheumatoid arthritis and vitamin D receptor.^16^ A complete description of the search strategy employed in this review is provided in Supplementary Table 1. The search was not limited to studies published in English.

### Eligibility Criteria

The eligibility criteria were based on the refined PICOS (Population, Intervention, Comparison, Outcomes, and Study Design) framework.^17^ Selected studies followed this criterion:

1. Participants: Adult patients diagnosed with rheumatoid arthritis.
2. Intervention/Exposure: None.
3. Comparison: Healthy controls.
4. Outcomes: The likelihood of developing rheumatoid arthritis in different VDR genes.
5. Study design: Observational studies (cohort, case-control, cross-sectional, etc.) comparing VDR genes in RA patients and healthy controls.

Exclusion criteria included the following:

1. Non-comparative (single-armed) studies of interventions.
2. Studies including <20 cases.
3. Abstract-only publications.
4. Conference and seminar presentations.
5 Duplicated records (if they provided the same outcome data for the same patient population).
6. Lack of relevant outcomes.
7. Non-original research (review articles, letter-to-editor, editorials, commentaries, etc.).
8. Study protocols without results.
9. Patients without rheumatoid arthritis.

### Study Selection and Data Extraction

Two independent reviewers screened the titles and abstracts of the retrieved citations. Full-text articles of potentially relevant studies were then assessed for eligibility.^18^ Discrepancies were resolved through discussion or by consulting a third reviewer.

A data sheet was structured based on available data from included studies. Data related to study characteristics (author, year, country, study design), patient characteristics (age, gender), rheumatoid arthritis data (duration, classification criteria, activity score, and remission), outcomes (as specified below), and likelihood of bias assessment were extracted. Data were extracted from the original studies. The primary outcome was the likelihood of RA in each VDR gene polymorphism: ApaI (rs7975232),^19^ BsmI (rs1544410),^20^ TaqI (rs731236),^20^ and FokI (rs2228570).^21^ For each polymorphism, 5 models were investigated: dominant model, recessive model, allelic model, aa vs. AA model, and Aa vs. AA model. Secondary outcomes included identification of effect modifiers through subgroup and meta-regression analyses.

### Methodological Quality Assessment

The methodological quality of included studies was assessed using the Newcastle Ottawa Scale (NOS) for case-control studies.^22^ The quality was assessed over 3 domains: selection (maximum 4 points), comparability (maximum 2 points), and exposure (maximum 4 points). Studies scoring 7-9 points were considered high quality, those scoring 4-6 points were considered fair quality, and studies scoring ≤3 points were classified as poor quality.

### Statistical Analysis

All statistical analyses were performed using Stata, version 18 (StataCorp LLC; College Station, TX, USA). Pairwise meta-analyses were conducted using the metan package,^23^ and meta-regression analyses were conducted using the “metareg” function.^24^ For binary outcomes, pooled ORs with corresponding 95% CIs were calculated to compare RA patients with healthy controls. Random-effects models were applied to account for anticipated clinical and methodological heterogeneity across studies.^25^ Separate analyses were performed for each VDR gene and model selection.

Subgroup analyses were performed to evaluate potential effect modifiers, including classification criteria, country of investigation, ethnicity, and presence of bone erosion.^26^ Meta-regression analyses were subsequently conducted to identify determinants of RA occurrence across genetic models.^27^ The examined covariates included classification criteria, ethnicity, bone erosion status, female-to-male ratio, age difference between RA and control groups, disease activity score, remission status, and disease duration. The female-to-male ratio and age difference between groups were included as covariates to account for potential differences in demographic characteristics between RA patients and controls. For meta-regression analyses, dummy variables were created, and the reference category was selected based on the largest sample size to ensure stable comparisons. Multicollinearity among covariates was assessed using variance inflation factors (VIFs), with VIF >5 indicating substantial multicollinearity; variables exceeding this threshold were excluded from the model.^28^

Statistical heterogeneity was assessed using the I² statistic, with values greater than 50% indicating substantial heterogeneity. Model fit for meta-regression analyses was evaluated using the *R*² statistic.^29^ Sensitivity analyses were performed to evaluate the robustness of the findings by sequentially omitting individual studies.^30^ Galbraith plots were inspected to identify potential outliers; identified data points were rechecked for accuracy and excluded only when confirmed to be valid outliers.^31^ Publication bias was assessed through visual inspection of funnel plots and formal tests for funnel plot asymmetry, including Egger’s regression test, Begg’s rank correlation test, and contour-enhanced funnel plots for binary outcomes.^32^

## Results

### Literature Review Results

**Figure 1.**
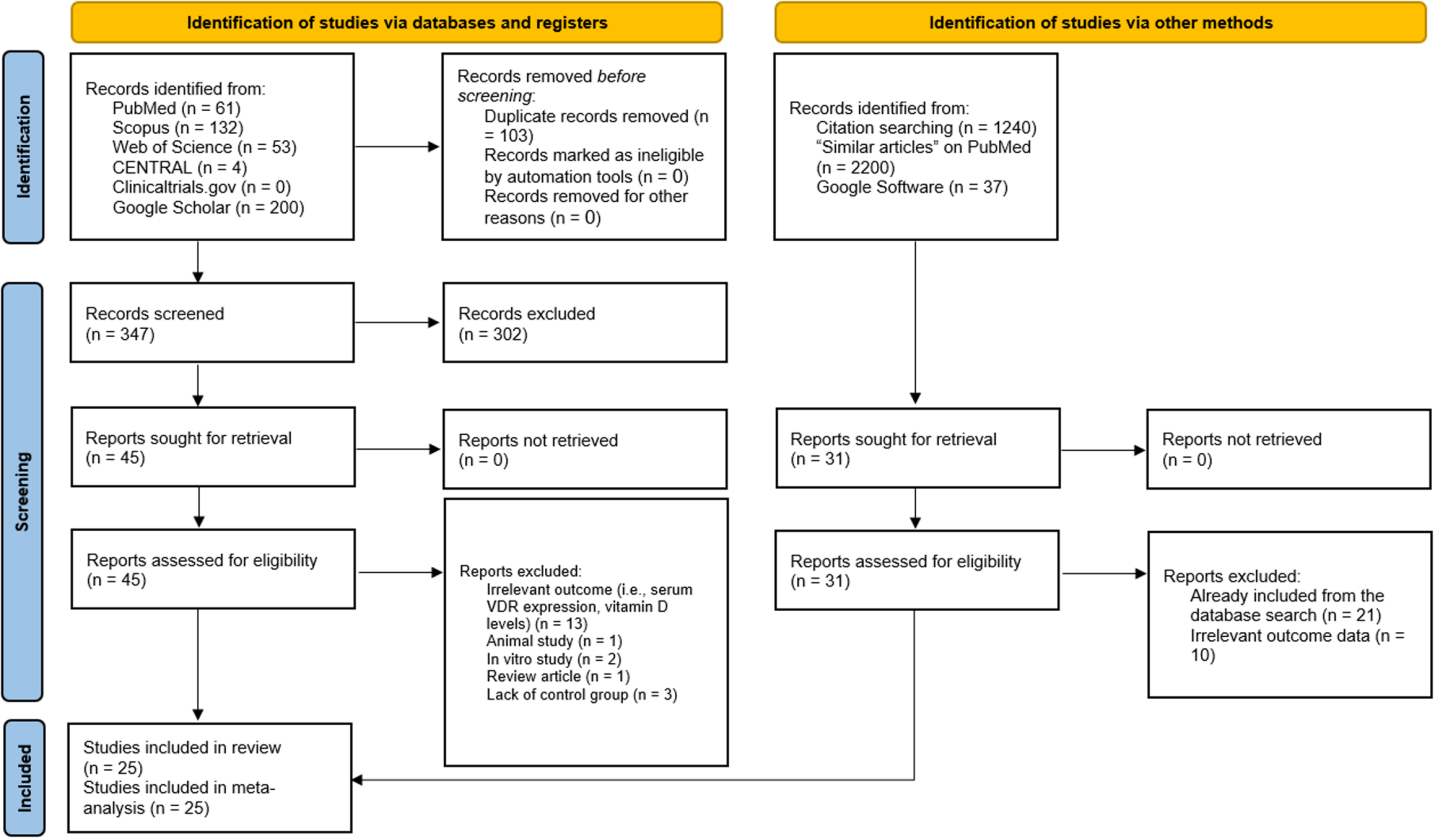
PRISMA flow diagram showing the results of the literature search.

A summary of the results of the literature search and screening processes is illustrated in Figure 1. The literature search yielded 450 citations. Following the removal of 103 duplicates through EndNote software (Version 8, USA), 347 articles were screened, of which 302 were excluded during the title and abstract screening phases. All full texts were found, resulting in 45 articles eligible for full-text screening. Articles were excluded for the following reasons: irrelevant outcome data (n = 13), animal study (n = 1), in vitro study (n = 2), review article (n = 1), and lack of a control group (n = 3). Finally, 25 case-control studies were analyzed.^6^^-^^12^^,^^33^^-^^50^

### Characteristics of Included Studies

The baseline characteristics of included studies and examined patients are summarized in Table 1. All included studies were case-control in design, most of which were conducted in Egypt (4 studies) followed by Pakistan (3 studies) and China (2 studies), respectively. In terms of ethnicity, Europeans accounted for the majority of investigated populations (9 studies) followed by Arabs (8 studies) and South Asian patients (4 studies), respectively. A total of 7595 patients were included, of whom 3711 were RA patients and the remaining 3884 constituted the healthy control group. The female-to-male ratio in the RA group was above one in all studies (ranging from 1.70 to 100), while in the control group a few studies reported a low female-to-male ratio of 0.27,^^46^^ 0.29,^12^ and 0.38.^39^

Regarding the classification of RA, 5 studies did not report a classification criterion. Meanwhile, the 1987 criteria set by the American Academy of Rheumatology (ACR) was the most frequently followed in 11 studies followed by the 2010 ACR/EULAR criteria in 8 studies. The disease duration ranged from 2.75 years to as high as 11.71 years, with remission rates ranging from 8.3 to 16.01%. Data regarding disease severity at baseline were scarcely reported. In terms of VDR gene identification, PCR-RFLP was the most frequently used method (17 studies).

### Methodological Quality Assessment

A summary of each study’s quality in each of the examined domains is provided in Table 2. Overall, all included studies had fair quality, with comparability being the most common domain where methodology was inadequate due to the absence of confounding adjustment through regression models or between-group matching.

### The Association Between Vitamin D Receptor Apal (rs7975232) and Rheumatoid Arthritis Occurrence

#### Dominant Model:

**Figure 2.**
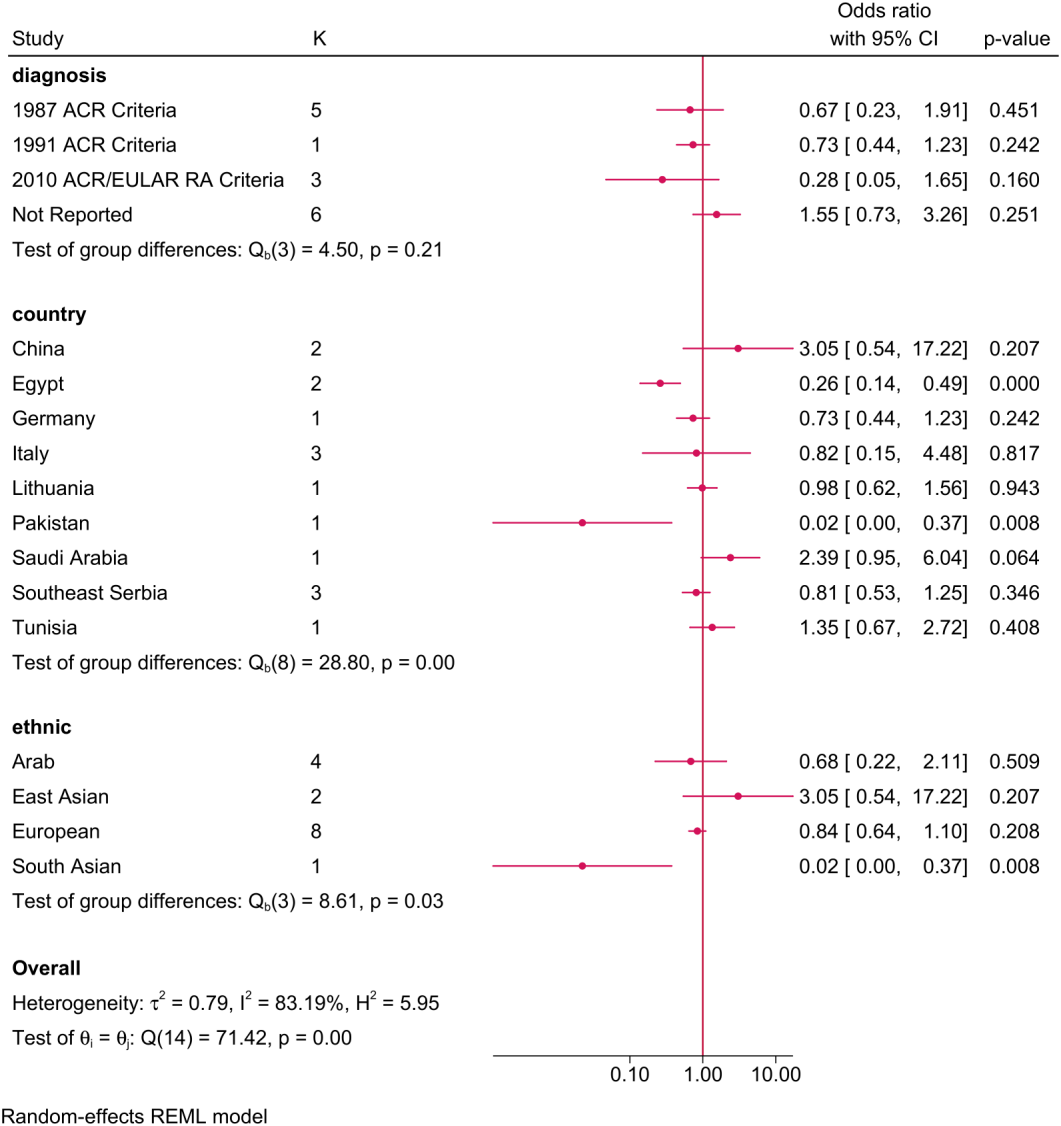
Forest plot showing the association between the VDR gene – Apal – (dominant model) between rheumatoid arthritis patients and healthy control, stratified by classification criteria, country, ethnicity, and bone erosion.

Country (*P* = .001) and ethnicity (*P* = .03) significantly altered the association between this model and the likelihood of developing RA, although the classification criteria of RA did not play a significant role (*P* = .21) (Figure 2). Both Egypt (2 studies, OR = 0.26; 95% CI: 0.14, 0.49) and Pakistan (1 study, OR = 0.02; 95% CI: 0.00, 0.37) exhibited a significant reduction in the likelihood of RA, while other countries showed a similar profile between this model and control. This reduction in the likelihood of RA was evident in South Asians (OR = 0.02; 95% CI: 0.00, 0.37) but not in other ethnic groups.

The meta-regression; however, showed that the 2010 ACR/EULAR classification criteria (coefficient=5.42, *P* = .007) increased the likelihood, while South Asian ethnicity (coefficient = −9.88, *P* = .0001) and F:M ratio (coefficient = −1.18, *P* = .008) demonstrated an opposite pattern in this model (Table 3).

#### Recessive Model:

**Figure 3.**
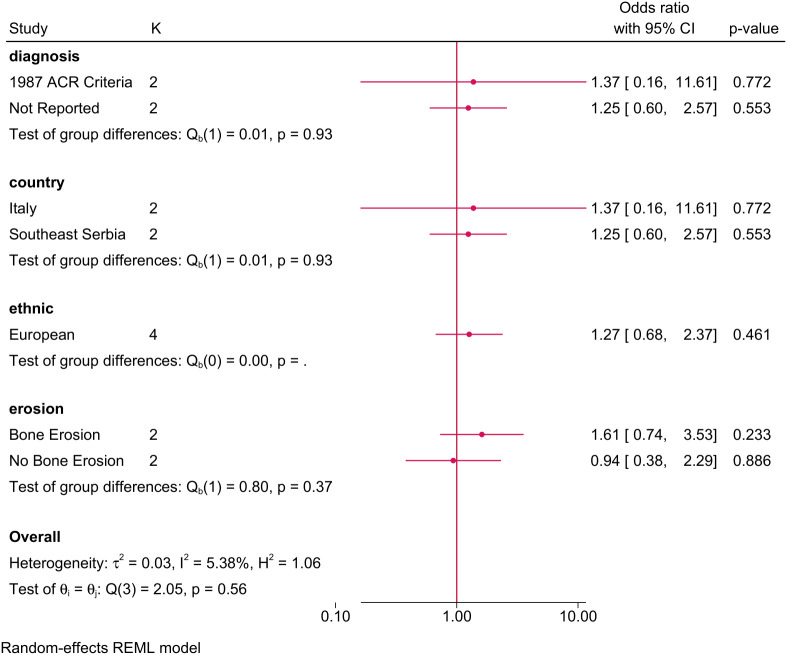
Forest plot showing the association between the VDR gene – Apal – (recessive model) between rheumatoid arthritis patients and healthy control, stratified by classification criteria, country, ethnicity, and bone erosion.

No significant association between the recessive model and RA was noted across different subgroups based on classification criteria (*P* = .93), country (*P* = .93), ethnicity, or bone erosion (*P* = .37) (Figure 3). However, the meta-regression showed that the 2010 ACR/EULAR criteria reduced the likelihood of RA (coefficient = −6.35, *P* = .023), while South Asian ethnicity (coefficient = 9.881, *P* = .0001) and F:M ratio further increased the likelihood of RA development.

#### Allelic Model:

Since all evidence was retrieved from Southeast Serbia (2 studies), lacking a classification criterion for RA, a subgroup meta-analysis was deemed unmeaningful.

#### aa vs AA Model:

No differences in the likelihood of RA between this model and controls were observed across all subgroups based on classification criteria (*P* = .88), country (*P* = .88), and bone erosion (*P* = .52) (Supplementary Figure 1). The meta-regression; however, showed that the 2010 ACR/EULAR criteria reduced the likelihood of RA (coefficient = −6.25, *P* = .032), while South Asian ethnicity (coefficient = 10.67, *P* = .0001) and F:M ratio (coefficient = 1.14, *P* = .015) showed positive associations with RA development.

#### Aa vs AA Model:

No differences in the likelihood of RA between this model and controls were observed across all subgroups based on classification criteria (*P* = .44), country (*P* = .44), and bone erosion (*P* = .72) (Supplementary Figure 2). The meta-regression; however, highlighted South Asian ethnicity as the sole determinant of the observed likelihood (coefficient = 6.67, *P* = .001) after accounting for other factors (Table 3).

### The Association Between Vitamin D Receptor Bsml (rs1544410) and **Rheumatoid Arthritis** Occurrence

#### Dominant Model:

No differences in the likelihood of RA between this model and controls were observed across all subgroups based on classification criteria (*P* = .32), countries (*P* = .62), and bone erosion (*P* = .53) (Supplementary Figure 3). The meta-regression; however, showed that the 1987 ACR criteria (coefficient = −1.094, *P* = .005) and F:M ratio, reduced the likelihood of RA, while patients’ age (coefficient = 0.13, *P* = .0001) revealed increased susceptibility (Table 4).

#### Recessive Model:

No differences in the likelihood of RA between this model and controls were observed across all subgroups based on classification criteria (*P* = .32), countries (*P* = .62), and bone erosion (*P* = .53) (Supplementary Figure 4). The meta-regression; however, showed that the 1987 ACR criteria (coefficient = −1.094, *P* = .005) and F:M ratio, reduced the likelihood of RA, while patients’ age (coefficient = 0.13, *P* = .0001) significantly increased disease susceptibility (Table 4).

#### Allelic Model:

No differences in the likelihood of RA between this model and controls were observed across all subgroups based on classification criteria (*P* = .78), countries (*P* = .95), and bone erosion (*P* = .31) (Supplementary Figure 5). The meta-regression; however, showed that the use of the 2010 ACR/EULAR (coefficient=-1.33; *P* = .0001) and 1987 ACR criteria (coefficient= -3.10; *P* = .0001) as well as F/M ratio (coefficient= -0.730; *P* = .0001) reduced RA likelihood, while patients’ age (coefficient=0.452; *P* = .0001) was conversely associated with RA (Table 4).

#### bb vs BB Model:

No differences in the likelihood of RA between this model and controls were observed across all subgroups based on classification criteria (*P* = .67), countries (*P* = .74), and bone erosion (*P* = .48) (Supplementary Figure 6). The meta-regression; however, showed that the 1987 ACR criteria (coefficient = 1.60; *P* = .002) and F/M ratio (coefficient = 0.228; *P* = .001) increased the likelihood of RA, while patients’ age (coefficient = −0.205; *P* = .0001) showed a protective effect (Table 4).

#### Bb vs BB Model:

RA’s classification criteria (*P* = .07) and bone erosion (*P* = .06) emerged as nearly significant effect modifiers of the association between this model and RA (Supplementary Figure 7). The meta-regression; however, showed that F:M ratio (coefficient = −0.018, *P* = .036) and patients’ age (coefficient = −0.111, *P* = .006) significantly reduced the likelihood of RA in this model (Table 4).

### The Association Between Vitamin D Receptor Taql (rs731 236) and **Rheumatoid Arthritis** Occurrence

#### Dominant Model:

No differences in the likelihood of RA between this model and controls were observed across all subgroups based on classification criteria (*P* = .91), countries (*P* = .96), and bone erosion (*P* = .76) (Supplementary Figure 8). The meta-regression, however, showed that 2010 ACR/EULAR criteria (coefficient = −2.759, *P* = .0013) reduced the likelihood, while F:M ratio (coefficient = 0.654, *P* = .007) showed a positive trend in this model (Table 5).

#### Recessive Model:

No differences in the likelihood of RA between this model and controls were observed across all subgroups based on classification criteria (*P* = .91), countries (*P* = .96), and bone erosion (*P* = .76) (Supplementary Figure 9). The meta-regression model failed to identify any significant determinants of the likelihood of RA after accounting for all examined variables (Table 5).

#### Allelic Model:

No differences in the likelihood of RA between this model and controls were observed across all subgroups based on classification criteria (*P* = .54), countries (*P* = .54), and bone erosion (*P* = .41) (Supplementary Figure 10). The meta-regression model failed to identify any significant determinants of the likelihood of RA after accounting for all examined variables (Table 5).

#### tt vs TT Model:

No differences in the likelihood of RA between this model and controls were observed across all subgroups based on classification criteria (*P* = .46), countries (*P* = .70), and bone erosion (*P* = .71) (Supplementary Figure 11). The meta-regression model failed to identify any significant determinants of the likelihood of RA after accounting for all examined variables (Table 5).

#### Tt vs TT Model:

**Figure 4.**
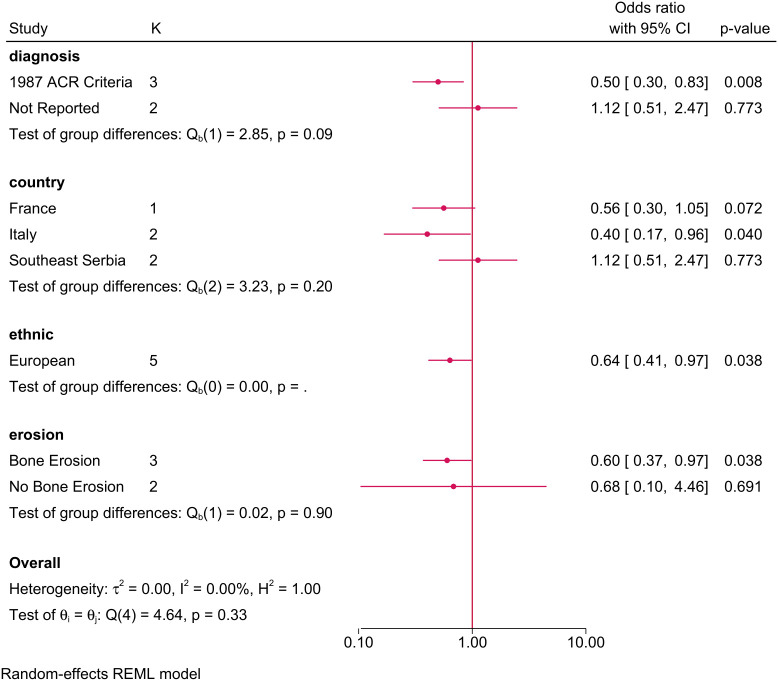
Sensitivity analysis of the risk of rheumatoid arthritis in the VDR gene – Taql (Tt vs. TT model).

Although the subgroup analysis failed to identify any significant likelihood modifiers in this model, a significant reduction in the likelihood of RA was observed in the following subgroups: 1987 ACR criteria (3 studies, OR = 0.50; 95% CI: 0.30, 0.83), Italy (2 studies, OR = 0.40; 95% CI: 0.17, 0.96), and patients with bone erosion (3 studies, OR = 0.60; 95% CI: 0.37, 0.97) (Figure 4). The meta-regression, however, identified South Asian ethnicity (coefficient = 4.98, *P* = .015) as the sole determinant of the observed likelihood (Table 5).

### The Association Between Vitamin D Receptor Fokl (rs2228570) and **Rheumatoid Arthritis** Occurrence

#### Dominant Model:

**Figure 5.**
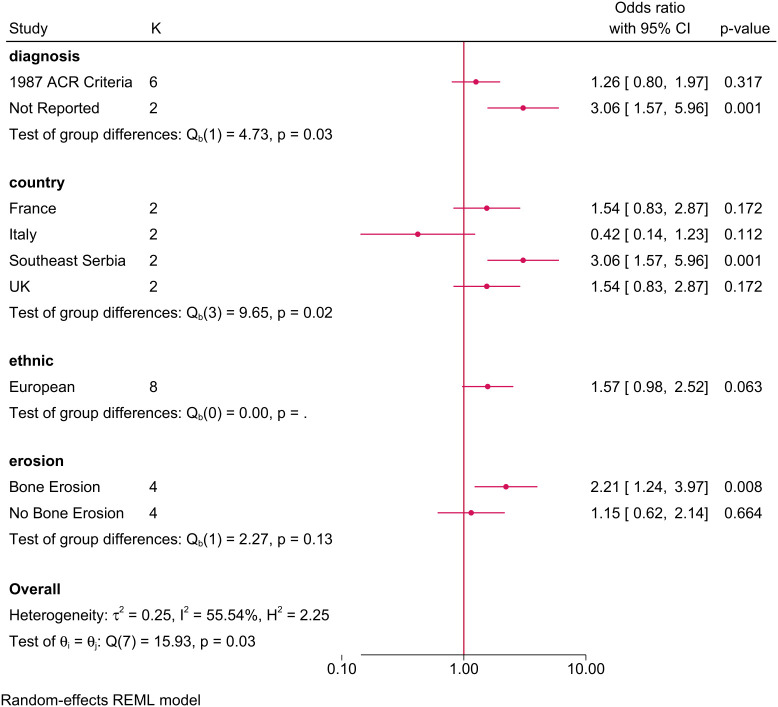
Forest plot showing the association between the VDR gene – Fokl – (dominant model) between rheumatoid arthritis patients and healthy control, stratified by classification criteria, country, ethnicity, and bone erosion.

RA’s classification criteria (*P* = .03) and country (*P* = .02) emerged as the sole effect modifiers of the likelihood of RA in this model. Specifically, a significant increase in the likelihood of RA compared to controls was observed only in patients from Serbia (2 studies, OR = 3.06; 95% CI: 1.57, 5.96) and those with bone erosion (4 studies, OR = 2.21; 95% CI: 1.24, 3.97) (Figure 5). Other subgroups showed no significant likelihood. Due to multicollinearity, the meta-regression findings were inconclusive (Table 6).

#### Recessive Model:

**Figure 6.**
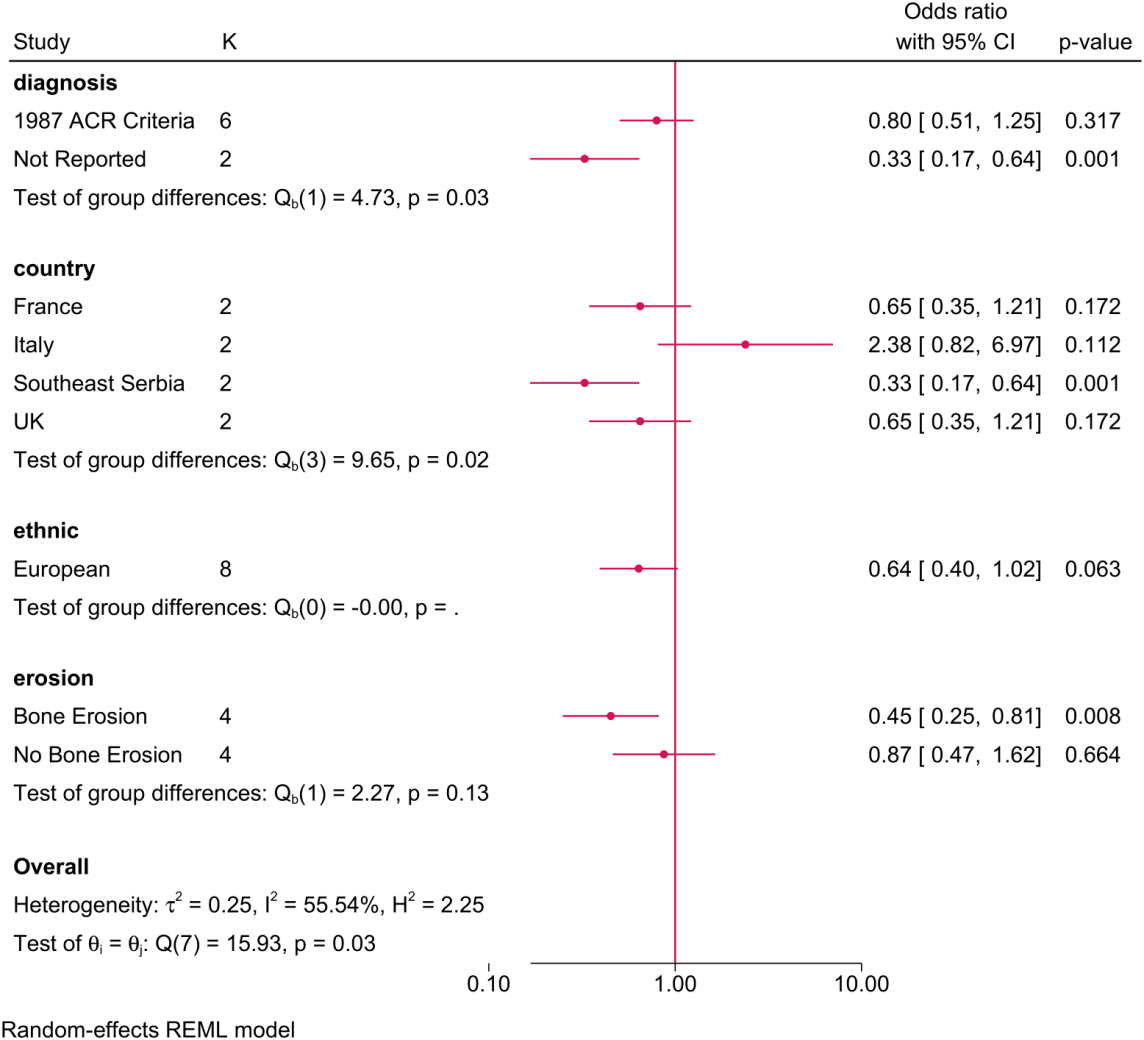
Forest plot showing the association between the VDR gene – Fokl – (recessive model) between rheumatoid arthritis patients and healthy control, stratified by classification criteria, country, ethnicity, and bone erosion.

RA’s classification criteria (*P* = .03) and country (*P* = .02) emerged as the sole effect modifiers of the likelihood of RA in this model. Specifically, a significant reduction in the likelihood of RA compared to controls was observed only in patients from Serbia (2 studies, OR = 0.33; 95% CI: 0.17, 0.64) and those with bone erosion (4 studies, OR = 0.45; 95% CI: 0.25, 0.81) (Figure 6). Due to multicollinearity, the meta-regression findings were inconclusive (Table 6).

#### Allelic Model:

**Figure 7.**
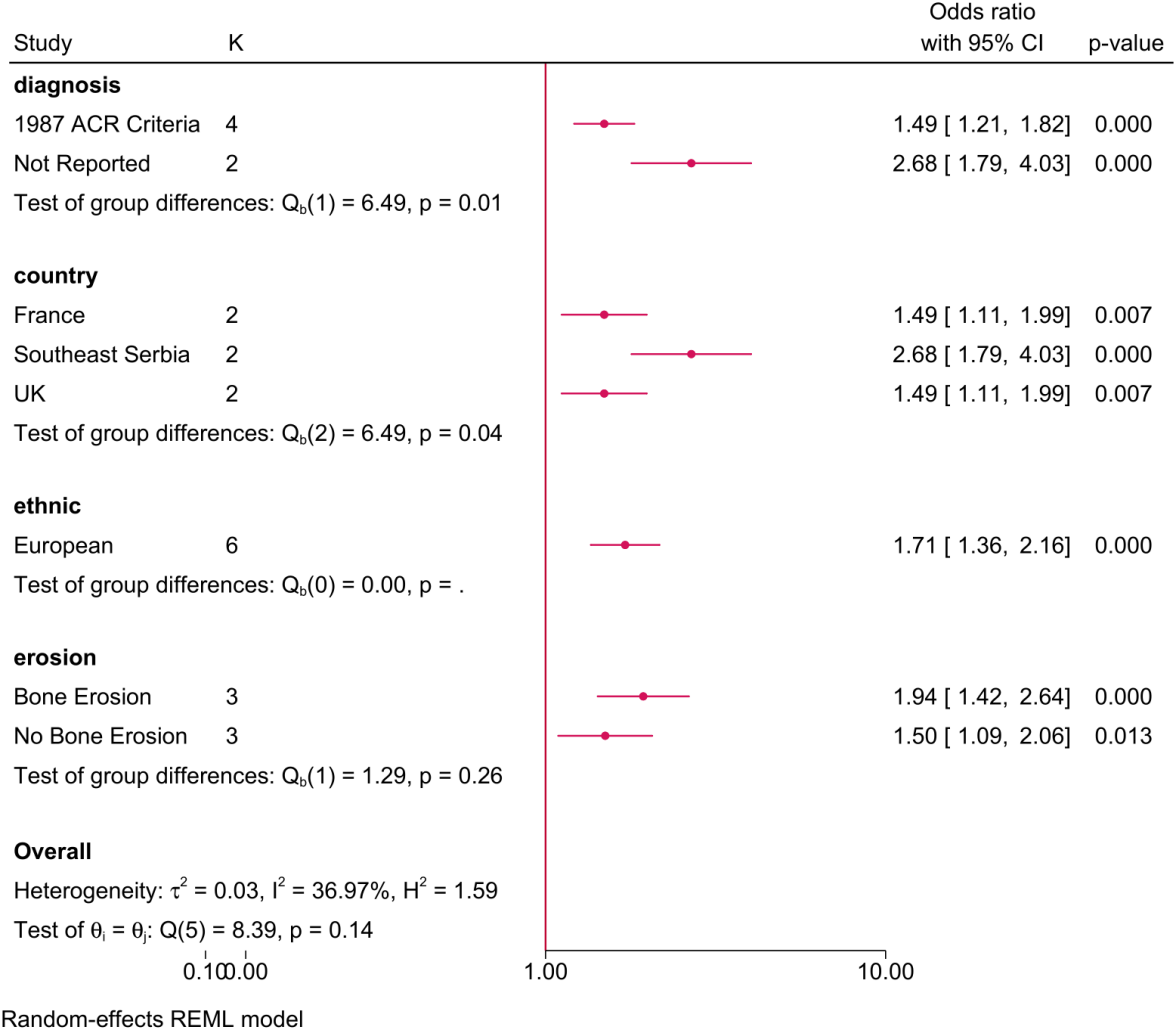
Forest plot showing the association between the VDR gene – Fokl – (allelic model) between rheumatoid arthritis patients and healthy control, stratified by classification criteria, country, ethnicity, and bone erosion.

Country-specific (*P* = .04) and classification criteria-based (*P* = .01) differences in the likelihood of RA were observed in this model (Figure 7). All subgroups exhibited an increased likelihood of RA in this model, but it was most pronounced in patients from Serbia (2 studies, OR = 2.68; 95% CI: 1.79, 4.03) and in those with bone erosion (3 studies, OR = 1.94; 95% CI: 1.42, 2.64). The meta-regression; however, identified F:M ratio as the sole determinant of the observed likelihood (coefficient = −1.38, *P* = .035) (Table 6).

#### ff vs FF Model:

**Figure 8.**
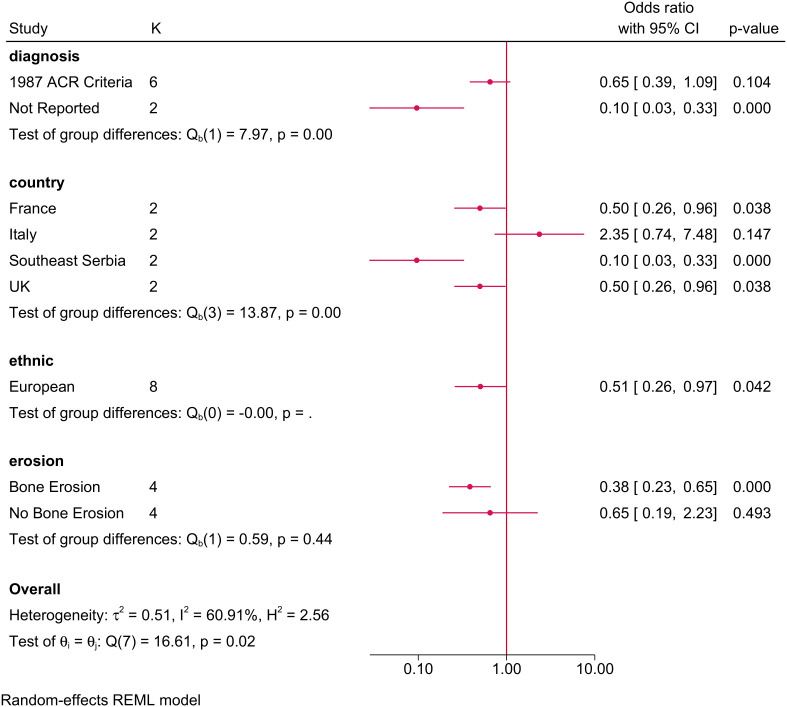
Forest plot showing the association between the VDR gene – Fokl – (ff vs. FF model) between rheumatoid arthritis patients and healthy control, stratified by classification criteria, country, ethnicity, and bone erosion.

Country-specific (*P* = .001) and classification criteria-based (*P* = .001) differences in the likelihood of RA were observed in this model (Figure 8). Specifically, patients from France, Serbia, and the UK showed a decreased likelihood of RA, while Italy showed a similar profile between RA and controls. Noteworthy, the likelihood of RA was reduced in patients with bone erosion (4 studies, OR = 0.38; 95% CI: 0.23, 0.65), while those without bone erosion showed no difference in the likelihood of RA development. The meta-regression, however, identified F:M ratio as the sole determinant of the observed likelihood (coefficient = 0.893, *P* = .034) (Table 6).

#### Ff vs FF Model:

**Figure 9.**
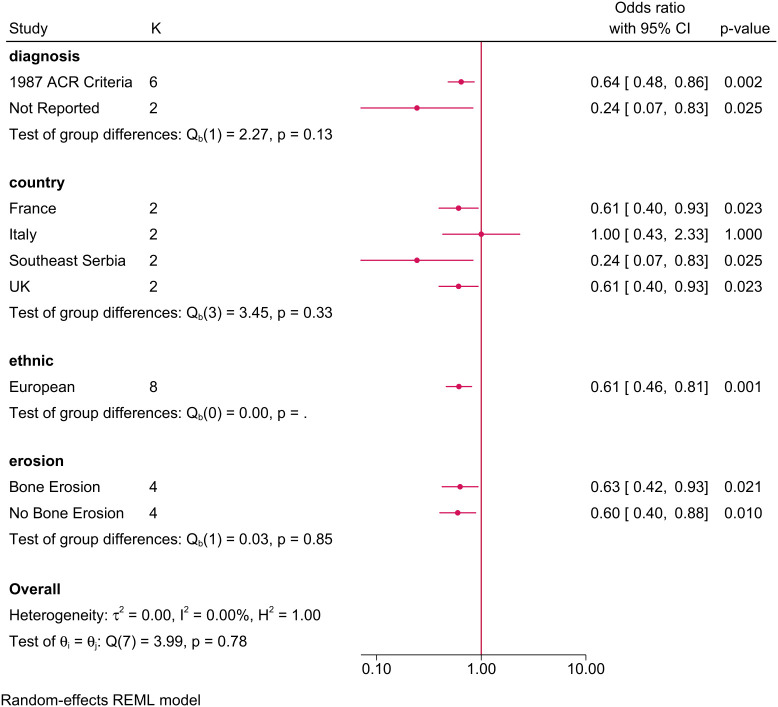
Forest plot showing the association between the VDR gene – Fokl – (Ff vs. FF model) between rheumatoid arthritis patients and healthy control, stratified by classification criteria, country, ethnicity, and bone erosion.

No differences in the likelihood of RA between this model and controls were observed across all subgroups based on classification criteria (*P* = .13), country (*P* = .33), or bone erosion (*P* = .85). Specifically, patients with and without bone erosion exhibited a similar reduction in the likelihood of developing RA compared to controls. However, although France, the UK, and Serbia showed reduced likelihood, Italy exhibited no difference in RA susceptibility (Figure 9). The meta-regression model failed to identify any significant determinants of the likelihood of RA after accounting for all examined variables (Table 6).

## Discussion

This meta-analysis expands upon prior work by shifting the focus from merely exploring associations between VDR polymorphisms and RA to identifying moderators of these associations. The findings demonstrate that ethnicity, bone erosion, and classification criteria significantly modulate the relationship between VDR polymorphisms and RA, addressing critical gaps identified in previous studies.

### Main Findings

The findings highlight significant ethnic variations, with South Asians showing reduced RA likelihood for ApaI and BsmI polymorphisms, while African populations displayed increased likelihood for BsmI. These results align with the growing recognition of genetic heterogeneity across populations and emphasize the need for studies that account for diverse genetic backgrounds. Such findings are critical for advancing precision medicine approaches, particularly in regions with underrepresented populations.

The classification criteria for RA also emerged as a significant moderator. Studies using the 2010 ACR/EULAR criteria exhibited distinct patterns compared to those employing the 1987 ACR criteria. These differences highlight the importance of standardized classification methodologies in genetic research, as variability in criteria can influence the observed associations and their interpretation.

Bone erosion, a hallmark of RA severity,^41^ was another critical moderator identified in this study. The presence of bone erosion significantly influenced the association between VDR polymorphisms and RA likelihood, suggesting that genetic effects may be more pronounced in patients with advanced disease. This finding underscores the importance of integrating clinical severity markers into genetic analyses to refine likelihood assessment and therapeutic strategies.

### Comparison with Prior Evidence

Numerous systematic reviews and meta-analyses have previously examined the link between VDR polymorphisms and autoimmune diseases, including RA. For instance, the meta-analysis by Tizaoui et al.^51^ confirmed associations between TaqI and FokI polymorphisms and RA susceptibility, noting significant heterogeneity driven by ethnicity and study design. Similarly, a study by Lee et al^52^ found that the F allele of the FokI polymorphism increased RA likelihood in Europeans (OR = 1.502, 95% CI = 1.158-1.949)​. These findings align with the current analysis, which underscores the moderating role of ethnicity, particularly in the observed protective effect of the ApaI polymorphism in South Asians.

In addition, the systematic review by Bagheri-Hosseinabadi et al^5^ noted that the functional implications of these polymorphisms varied significantly by population, with BsmI polymorphism increasing RA likelihood in Africans while TaqI polymorphism was protective in Arab populations. The findings are consistent with this population-specific variability, further substantiating the need for stratified analyses in genetic association studies.

### Clinical Relevance of Moderator-Based Analyses

Although numerous studies and meta-analyses have examined the association between vitamin D receptor polymorphisms and rheumatoid arthritis,^5^^,^^51^^-^^54^ their clinical applicability has remained limited due to inconsistent and population-dependent findings. By focusing on moderators rather than direct associations, the present study provides clinically relevant context for interpreting this heterogeneity.

Identification of ethnicity, classification criteria, and bone erosion as effect modifiers helps explain why prior studies have reported conflicting results and underscores that genetic susceptibility signals may not be uniform across patient populations or disease phenotypes. From a clinical perspective, this emphasizes that genetic findings related to VDR polymorphisms should be interpreted cautiously and within appropriate population and disease-severity contexts rather than being applied universally.

Moreover, the observed modification by bone erosion suggests that genetic effects may differ according to structural disease severity, which is clinically relevant when considering prognosis-oriented research or studies linking VDR variants to skeletal outcomes in rheumatoid arthritis. While these findings do not currently support routine genetic testing in clinical practice, they provide a framework for future studies aimed at integrating genetic markers with clinical features to improve patient stratification and outcome prediction.

Importantly, the findings of the present study should not be interpreted as supporting changes in routine clinical management of patients with rheumatoid arthritis. The subgroup and moderator effects identified reflect population-level patterns derived from aggregated data and are not intended to guide individual patient diagnosis, prognosis, or treatment decisions. At present, there is insufficient evidence to justify routine vitamin D receptor genotyping in clinical practice.

From a health-economics perspective, the modest and context-dependent effects observed in this analysis suggest that widespread implementation of VDR polymorphism testing would be unlikely to be cost-effective. Formal cost-effectiveness analyses were beyond the scope of this study and would require prospective evaluation integrating genetic information with treatment response and clinical outcomes.

### Vitamin D Levels and Disease Activity

Beyond genetic polymorphisms, serum vitamin D levels have been implicated in RA activity. Lin et al^53^ reported a significant inverse relationship between serum 25-hydroxyvitamin D (25OHD) levels and Disease Activity Score in 28 joints (DAS28) (r = −0.13, 95% CI = −0.16 to −0.09)​. Similarly, Harrison et al^55^ highlighted the immunomodulatory effects of vitamin D in promoting tolerogenic regulatory T cells and suppressing pro-inflammatory Th1 responses​. These mechanisms may explain the observed associations in our meta-regression analyses, particularly in regions with high prevalence of vitamin D deficiency.

### Implications of Classification Criteria and Bone Erosion

The influence of classification criteria, such as the 1987 ACR versus 2010 ACR/EULAR guidelines, highlights an important source of methodological heterogeneity in rheumatoid arthritis research. As noted in prior meta-analyses, inconsistent diagnostic frameworks can lead to conflicting findings in genetic association studies by altering the underlying disease spectrum captured across studies.^56^​

The 1987 ACR criteria were developed to classify established rheumatoid arthritis and emphasize clinical manifestations such as persistent synovitis, radiographic changes, and longer disease duration. In contrast, the 2010 ACR/EULAR criteria were designed to improve sensitivity for early and serologically active disease by incorporating autoantibody status and acute-phase reactants. Consequently, studies applying the 2010 criteria may include patients at earlier disease stages with less structural damage, whereas those using the 1987 criteria are more likely to capture patients with advanced disease phenotypes.

In the current analysis, classification criteria emerged as a significant moderator in several genetic models, suggesting that variation in case definition influences the observed associations between VDR polymorphisms and rheumatoid arthritis risk. In parallel, the inclusion of bone erosion as a clinical moderator revealed stronger associations for the FokI polymorphism in patients with erosive disease (OR = 2.21, 95% CI = 1.24-3.97), indicating that genetic effects may be more evident in patients with greater structural severity. Together, these findings underscore the importance of accounting for both disease definition and severity when interpreting genetic susceptibility in rheumatoid arthritis.

### Study Limitations

The existing literature has several limitations that may have influenced our findings. A major issue is the widespread lack of adjusted regression models to account for confounding factors such as RA disease activity, duration, comorbid osteoporosis or bone erosion, BMD, and vitamin D levels. These factors can interact with VDR polymorphisms and confound the observed associations, obscuring the independent effects of these genetic variants. Additionally, most studies focus on RA occurrence, with limited exploration of how VDR polymorphisms impact clinical outcomes, such as disease severity, progression, or response to treatment. Understanding whether specific VDR genotypes influence disease trajectory or the efficacy of vitamin D supplementation remains an important but underexplored area.

### Mechanistic Insights and Future Directions

The immunomodulatory role of vitamin D, mediated through VDR, is well-established in regulating T cell differentiation and cytokine production. However, as noted by Usategui-Martin et al,^54^ the response to vitamin D supplementation may vary based on genetic polymorphisms, with TaqI and FokI associated with better outcomes. This highlights the potential for personalized interventions based on genetic profiles. Future research should prioritize longitudinal studies to assess the causal relationships between VDR polymorphisms, vitamin D levels, and RA outcomes. Additionally, exploration of gene-environment interactions, particularly in populations with high vitamin D deficiency, is warranted. Finally, the standardization of classification criteria is of utmost importance to reduce heterogeneity across studies.

By incorporating evidence from multiple systematic reviews and meta-analyses, this study emphasizes the critical role of moderators in shaping the relationship between VDR polymorphisms and RA. These findings not only advance the understanding of genetic contributions to RA but also pave the way for tailored therapeutic approaches that consider population-specific genetic and environmental factors.

#### Data Availability Statement:

The data that support the findings of this study are available on request from the corresponding author.

#### Artificial Intelligence Usage Statement:

The authors declared that this study used ChatGPT to improve language, edit grammar, and generate content during the preparation of the manuscript. The content was revised, validated, and edited by the authors who take full responsibility for the accuracy of the provided content.

#### Peer-review:

Externally peer-reviewed.

#### Author Contributions:

Concept – W.L., S.X.; Design –W.L., S.X.; Supervision – S.X.; Resources – S.X.; Materials – W.L., S.X.; Data Collection and/or Processing – S.X.t; Analysis and/or Interpretation – W.L.; Literature Search – W.L.; Writing – W.L., S.X.r; Critical Review – W.L., S.X.

#### Declaration of Interests:

The authors have no conflicts of interest to declare.

#### Funding:

The authors declare that this study received no financial support.

## Supplementary Materials

Supplementary Material

## Figures and Tables

**Table 1.** A Summary of the Characteristics of Included Studies Reporting VDR Polymorphisms in Rheumatoid Arthritis Patients

**Author (YOP)**	**Design**	**Country**	**Ethnicity**	**Sample (Case/Control)**		**Age**		**F/M Ratio**		**Genotyping Method**	**RA Characteristics**			
				**RA**	**HC**	**RA**	**HC**	**RA**	**HC**		**DAS; M (SD)**	**Duration (year)**	**Remission**	**Diagnosis**
Despotović^^33^^	CC	Southeast Serbia	European	143	105	57.04 (12.48)	56 (16.59)	3.93	2.09	PCR-RFLP	–	–	–	Not reported
Di Spigna^6^	CC	Italy	European	40	40	57.5 (12.5)	40.3 (11.3)	3	100	PCR-RFLP	–	8.75	–	1987 ACR criteria
Idriss^37^	CC	Egypt	Arab	70	61	45.72 (11.6)	35.95 (11.9)	9	19.33	PCR-RFLP	–	–	–	2010 ACR/EULAR RA criteria
John^38^	CC	Pakistan	South Asian	100	100	44.2 (-)^†^	43 (-)^†^	1.7	2.23	PCR-RFLP	–	–	–	2010 ACR/EULAR RA criteria
Punceviciene^^44^^	CC	Lithuania	European	206	180	55.01 (11.08)	53.15 (10.68)	8.36	9.59	qPCR - VDR Genotyping	High (27.18%) Moderate (47.57%) Low (9.22)	11.71	16.01	2010 ACR/EULAR RA criteria
Garcia-Lozano^^34^^	CC	Spain	European	120	200	–	–	–	–	PCR-RFLP	–	–	–	Not reported
Ghelani^35^	CC	UK	European	271	299	–	–	3.77	3.44	PCR-RFLP	–	–	–	1987 ACR criteria
Goertz^^9^^	CC	Germany	European	62	40	57.4 (14.8)	53.2 (15.2)	–	–	PCR	–	10.41	–	1991 ACR criteria
Hitchon^^36^^	CC	USA	North American	448	705	47 (15)	35 (12)	4.53	1.44	Sequenom	–	–	–	1987 ACR criteria
Hussien^^50^^	CC	Egypt	Arab	200	150	57.3 (3.9)	57.1 (3.8)	100	​	PCR-RFLP	4.09	1.25	–	1987 ACR criteria
John^7^	CC	Pakistan	South Asian	100	100	44.2 (-)^†^	43 (-)^†^	1.70	2.23	ARMS-PCR	–	–	–	2010 ACR/EULAR RA criteria
Karray^^39^^	CC	Tunisia	Arab	108	152	39.5 (13)	41.3 (9)	4.14	0.38	PCR-RFLP	High (29.6%) Moderate (49%) Low (12.9%)	8.69	8.3	1987 ACR criteria
Khoja^^40^^	CC	Saudi Arabia	Arab	37	40	49.4 (-)^†^	45.1 (-)^†^	–	–	PCR-RFLP	–	–	–	Not reported
Lee^^41^^	CC	Korea	East Asian	157	120	45.77 (-)^†^	38.14 (-)^†^	7.26	–	PCR-RFLP	–	–	–	1987 ACR criteria
Maalej^^43^^	CC	France	European	100	100	–	–	6.69	9	PCR-RFLP	–	–	–	1987 ACR criteria
Mukhtar^^11^^	CC	Pakistan	South Asian	300	316	38.92 (-)^†^	52.3 (-)^†^	3.23	2.16	PCR-RFLP	–	7.27	–	2010 ACR/EULAR RA criteria
Rass^45^	CC	Hungary	European	64	40	51.2 (23.2)	46.7 (19.4)	7	9	PCR	–	–	–	1987 ACR criteria
Saad^^47^^	CC	Egypt	Arab	105	80	42.71 (12.07)	–	5.56	6.27	PCR-RFLP	–	2.85	–	1987 ACR criteria
Tizaoui^^12^^	CC	Tunisia	Arab	106	153	51.66 (5.7)	44.64 (7.93)	4.05	0.29	PCR	High (29.24%) Moderate (49.05%) Low (13.2)	6.45	8.4	1987 ACR criteria
Huang^^49^^	CC	China	East Asian	236	220	44.31	31.12	–	–	PCR-MassARRAY	–	–	–	Not reported
Mosaad^^8^^	CC	Egypt	Arab	128	150	46.91	40	4.12	–	PCR-RFLP	5.33	10.33	–	2010 ACR/EULAR RA criteria
Shukla^^48^^	CC	India	South Asian	112	125	–	–	–	–	PCR-RFLP	–	–	–	1987 ACR criteria
Rodriguez-Carrio^^46^^	CC	Spain	European	194	88	53.87 (19-87.81)*	53.25 (27-83)*	3.97	0.27	TaqMan	3.7	2.75	–	2010 ACR/EULAR RA criteria
Mahmoud^^10^^	CC	Jordan	Arab	184	200	–	–	–	–	PCR-RFLP	–	–	–	2010 ACR/EULAR RA criteria
Li (2013)^^42^^	CC	China	East Asian	120	120	44 (-)^†^	46 (-)^†^	–	–	PCR-RFLP	–	–	–	Not reported

ACR, American Academy of Rheumatology; CC, case-control; DAS, disease activity score; F/M, female-to-male ratio; M, mean; RA, rheumatoid arthritis; SD, standard deviation; YOP, year of publication.

*Data in age are presented as mean (standard deviation) except for this value where the mean (range) is reported instead.

†The mean values are reported without their standard deviations.

**Table 2.** The methodological Quality of Included Case-Control Studies Measured by the Newcastle–Ottawa Scale

**Author (YOP)**	**Selection**				**Comparability**		**Exposure**			**Overall Rating**
	**Is the Case Definition Adequate?**	**Representativeness of the Cases**	**Selection of Controls**	**Definition of Controls**	**Main Confounding Control**	**Additional Confounding Control**	**Ascertainment of Exposure**	**Same Method of Ascertainment for Cases and Controls**	**Non-Response Rate**	
Despotović^^33^^	Yes	Yes	Yes	Yes	No	No	No	Yes	Yes	Fair
Di Spigna^^6^^	Yes	Yes	Yes	Yes	No	No	Yes	Yes	Yes	Fair
Idriss^^37^^	Yes	Yes	Yes	Yes	No	No	Yes	Yes	Yes	Fair
John^^38^^	Yes	Yes	Yes	Yes	No	No	Yes	Yes	Yes	Fair
Punceviciene^^44^^	Yes	Yes	Yes	Yes	No	No	Yes	Yes	Yes	Fair
Garcia-Lozano^^34^^	Yes	Yes	Yes	Yes	No	No	No	Yes	Yes	Fair
Ghelani^^35^^	Yes	Yes	Yes	Yes	No	No	Yes	Yes	Yes	Fair
Goertz^^9^^	Yes	Yes	Yes	Yes	No	No	Yes	Yes	Yes	Fair
Hitchon^^36^^	Yes	Yes	Yes	Yes	No	No	Yes	Yes	Yes	Fair
Hussien^^50^^	Yes	Yes	Yes	Yes	No	No	Yes	Yes	Yes	Fair
John^^7^^	Yes	Yes	Yes	Yes	No	No	Yes	Yes	Yes	Fair
Karray^^39^^	Yes	Yes	Yes	Yes	No	No	Yes	Yes	Yes	Fair
Khoja^^40^^	Yes	Yes	Yes	Yes	No	No	No	Yes	Yes	Fair
Lee^^41^^	Yes	Yes	Yes	Yes	No	No	Yes	Yes	Yes	Fair
Maalej^^43^^	Yes	Yes	Yes	Yes	No	No	Yes	Yes	Yes	Fair
Mukhtar^^11^^	Yes	Yes	Yes	Yes	No	No	Yes	Yes	Yes	Fair
Rass^^45^^	Yes	Yes	Yes	Yes	No	No	Yes	Yes	Yes	Fair
Saad^^47^^	Yes	Yes	Yes	Yes	No	No	Yes	Yes	Yes	Fair
Tizaoui^^12^^	Yes	Yes	Yes	Yes	No	No	Yes	Yes	Yes	Fair
Huang^^49^^	Yes	Yes	Yes	Yes	No	No	No	Yes	Yes	Fair
Mosaad^^8^^	Yes	Yes	Yes	Yes	No	No	Yes	Yes	Yes	Fair
Shukla^^48^^	Yes	Yes	Yes	Yes	No	No	Yes	Yes	Yes	Fair
Rodriguez-Carrio^^46^^	Yes	Yes	Yes	Yes	No	No	Yes	Yes	Yes	Fair
Mahmoud^^10^^	Yes	Yes	Yes	Yes	No	No	Yes	Yes	Yes	Fair
Li^^42^^	Yes	Yes	Yes	Yes	No	No	No	Yes	Yes	Fair

**Table 3.** Meta-Regression Analysis of the Determinants of the Risk Between VDR Polymorphism (Apal (rs7975232)) and Rheumatoid Arthritis Occurrence

​	**Coefficient**	**SE**	**z**	***P***	**Low CI**	**High CI**
Dominant model							Diagnostic criteria (Reference group: Not reported)						
2010 ACR/EULAR RAcriteria	5.429	2.015	2.690	.007	1.480	9.378
1987 ACR criteria	−0.923	1.524	−0.610	.545	−3.909	2.064
Ethnicity (Reference group: European)						
Arab	1.544	1.606	0.960	.336	−1.603	4.691
South Asian	−9.881	2.713	−3.640	.000	−15.198	−4.563
F:M ratio (RA group; per point)	−1.188	0.446	−2.660	.008	−2.062	−0.314
Recessive model							Diagnostic criteria (Reference group: Not reported)						
2010 ACR/EULAR RA criteria	−6.352	2.797	−2.270	.023	−11.834	−0.870
Ethnicity (Reference group: European)						
Arab	−1.544	1.606	−0.960	.336	−4.691	1.603
South Asian	9.881	2.713	3.640	.000	4.563	15.198
F:M ratio (RA group; per point)	1.188	0.446	2.660	.008	0.314	2.062
Allelic m,odel							Diagnostic criteria (Reference group: Not reported)						
2010 ACR/EULAR RA criteria	0.032	0.889	0.040	.972	−1.711	1.775
Ethnicity (Reference group: European)						
South Asian	0.505	1.228	0.410	.681	−1.902	2.913
F:M ratio (RA group; per point)	−0.023	0.234	−0.100	.920	−0.482	0.435
aa vs. AA model							Diagnostic criteria (Reference group: Not reported)						
2010 ACR/EULAR RA criteria	−6.261	2.916	−2.150	.032	−11.976	−0.545
Ethnicity (Reference group: European)						
Arab	−1.539	1.635	−0.940	.347	−4.744	1.666
South Asian	10.670	2.826	3.780	.000	5.130	16.209
F:M ratio (RA group; per point)	1.148	0.471	2.440	.015	0.226	2.070
Aa vs. AA model							Diagnostic criteria (Reference group: Not reported)						
2010 ACR/EULAR RA Criteria	0.865	1.702	0.510	.612	−2.472	4.201
Ethnicity (Reference group: European)						
Arab	0.521	0.929	0.560	.575	−1.299	2.341
South Asian	6.675	2.034	3.280	.001	2.689	10.661
F:M ratio (RA group; per point)	−0.106	0.279	−0.380	.705	−0.654	0.442

ACR, American Academy of Rheumatology; F:M ratio, female-to-male ratio; HC, healthy control; RA, rheumatoid arthritis; SE, standard error; VDR, vitamin D receptor.

**Table 4.** Meta-Regression Analysis of the Determinants of the Risk Between VDR Polymorphism (Bsml (rs1544410)) and Rheumatoid Arthritis Occurrence

​	**Coefficient**	**SE**	**z**	***P***	**Low CI**	**High CI**
Dominant model							Diagnostic criteria (Reference group: Not reported)						
2010 ACR/EULAR RA criteria	−0.020	0.323	−0.060	.950	−0.653	0.613
1987 ACR criteria	−1.094	0.388	−2.820	.005	−1.855	−0.334
F:M ratio (RA group; per point)	−0.115	0.052	−2.190	.029	−0.218	−0.012
F:M ratio (HC group; per point)	0.011	0.006	2.050	.040	0.001	0.022
Age difference between RA and HC (per point)	0.138	0.038	3.610	.000	0.063	0.212
Recessive model							Diagnostic criteria (Reference group: Not reported)						
2010 ACR/EULAR RA criteria	0.020	0.323	0.060	.950	−0.613	0.653
1987 ACR criteria	1.094	0.388	2.820	.005	0.334	1.855
F:M ratio (RA group; per point)	0.115	0.052	2.190	.029	0.012	0.218
F:M ratio (HC group; per point)	−0.011	0.006	−2.050	.040	−0.022	−0.001
Age difference between RA and HC (per point)	−0.138	0.038	−3.610	.000	−0.212	−0.063
Allelic model							Diagnostic criteria (Reference group: Not reported)						
2010 ACR/EULAR RA criteria	−1.331	0.364	−3.660	.000	−2.045	−0.618
1987 ACR criteria	−3.101	0.495	−6.260	.000	−4.072	−2.131
F:M ratio (RA group; per point)	−0.730	0.137	−5.350	.000	−0.998	−0.463
F:M ratio (HC group; per point)	0.560	0.108	5.180	.000	0.348	0.772
Age difference between RA and HC (per point)	0.452	0.070	6.450	.000	0.315	0.590
bb vs. BB model							Diagnostic criteria (Reference group: Not reported)						
2010 ACR/EULAR RA criteria	−0.234	0.449	−0.520	.602	−1.113	0.645
1987 ACR criteria	1.602	0.517	3.100	.002	0.589	2.616
F:M ratio (RA group; per point)	0.228	0.068	3.340	.001	0.094	0.362
F:M ratio (HC group; per point)	−0.024	0.009	−2.750	.006	−0.042	−0.007
Age difference between RA and HC (per point)	−0.205	0.047	−4.380	.000	−0.296	−0.113
Bb vs. BB model							Diagnostic criteria (Reference group: Not reported)						
2010 ACR/EULAR RA criteria	−0.300	0.421	−0.710	.476	−1.125	0.525
1987 ACR criteria	0.842	0.485	1.730	.083	−0.110	1.793
F:M ratio (RA group; per point)	0.152	0.055	2.740	.006	0.043	0.261
F:M ratio (HC group; per point)	−0.018	0.009	−2.100	.036	−0.035	−0.001
Age difference between RA and HC (per point)	−0.111	0.041	−2.720	.006	−0.192	−0.031

ACR, American Academy of Rheumatology; F:M ratio, female-to-male ratio; HC, healthy control; RA, rheumatoid arthritis; SE, standard error; VDR, vitamin D receptor.

**Table 5.** Meta-Regression Analysis of the Determinants of the Risk Between VDR Polymorphism (Taql (rs731236)) and Rheumatoid Arthritis Cccurrence

​	**Coefficient**	**SE**	**z**	***P***	**Low CI**	**High CI**
Dominant model							Diagnostic criteria (Reference group: Not reported)						
2010 ACR/EULAR RA criteria	−2.759	1.115	−2.470	.013	−4.945	−0.574
1987 ACR criteria	0.264	0.530	0.500	.619	−0.775	1.303
F:M ratio (RA group; per point)	0.654	0.244	2.680	.007	0.175	1.132
F:M ratio (HC group; per point)	0.004	0.009	0.410	.682	−0.014	0.022
Age difference between RA and HC (per point)	0.010	0.090	0.110	.911	−0.166	0.187
Recessive model							Diagnostic criteria (Reference group: Not reported)						
2010 ACR/EULAR RA criteria	2.674	1.800	1.480	.138	−0.855	6.202
1987 ACR criteria	−0.600	0.793	−0.760	.449	−2.155	0.954
Ethnicity (Reference group: European)						
Arab	0.281	1.009	0.280	.781	−1.696	2.259
South Asian	0.239	2.509	0.100	.924	−4.679	5.157
F:M ratio (RA group; per point)	−0.643	0.394	−1.630	.103	−1.415	0.129
Allelic model							Diagnostic criteria (Reference group: Not reported)						
2010 ACR/EULAR RA criteria	0.630	1.168	0.540	.590	−1.659	2.918
1987 ACR criteria	0.033	0.429	0.080	.938	−0.807	0.874
Ethnicity (Reference group: European)						
South Asian	−0.281	1.291	−0.220	.828	−2.811	2.250
F:M ratio (RA group; per point)	−0.122	0.248	−0.490	.625	−0.609	0.365
tt vs. TT model							Diagnostic criteria (Reference group: Not reported)						
2010 ACR/EULAR RA criteria	3.551	1.969	1.800	.071	−0.308	7.411
1987 ACR criteria	−0.735	0.893	−0.820	.411	−2.486	1.016
Ethnicity (Reference group: European)						
Arab	0.448	1.084	0.410	.679	−1.676	2.572
South Asian	−0.048	2.655	−0.020	.986	−5.252	5.156
F:M ratio (RA group; per point)	−0.815	0.428	−1.900	.057	−1.654	0.024
Tt vs. TT model							Diagnostic criteria (Reference group: Not reported)						
2010 ACR/EULAR RA criteria	1.476	1.341	1.100	.271	−1.152	4.105
1987 ACR criteria	−0.285	0.666	−0.430	.669	−1.589	1.020
Ethnicity (Reference group: European)						
Arab	0.294	0.706	0.420	.678	−1.090	1.677
South Asian	4.984	2.056	2.420	.015	0.954	9.015
F:M ratio (RA group; per point)	−0.298	0.287	−1.040	.298	−0.861	0.264

ACR, American Academy of Rheumatology; F:M ratio, female-to-male ratio; HC, healthy control; RA, rheumatoid arthritis; SE, standard error; VDR, vitamin D receptor.

**Table 6.** Meta-Regression Analysis of the Determinants of the Risk Between VDR Polymorphism (Fokl (rs2228570)) and Rheumatoid Arthritis Occurrence

​	**Coefficient**	**SE**	**z**	***P***	**Low CI**	**High CI**
Dominant model						
F:M ratio (RA group; per point)	0.000	0.189	0.000	1.000	−0.371	0.371
Recessive model						
F:M ratio (RA group; per point)	0.000	0.189	0.000	1.000	−0.371	0.371
Allelic model							Diagnostic criteria (Reference group: Not reported)						
2010 ACR/EULAR RA criteria	1.652	1.619	1.020	0.308	−1.522	4.826
1987 ACR criteria	−0.080	1.515	−0.050	0.958	−3.049	2.890
F:M ratio (RA group; per point)	−1.384	0.657	−2.110	0.035	−2.672	−0.097
F:M ratio (HC group; per point)	0.465	0.303	1.540	0.125	−0.129	1.058
ff vs. FF model							Diagnostic criteria (Reference group: Not reported)						
2010 ACR/EULAR RA criteria	−1.300	2.854	−0.460	0.649	−6.895	4.294
1987 ACR criteria	0.646	2.638	0.250	0.806	−4.523	5.816
F:M ratio (RA group; per point)	0.893	0.422	2.120	0.034	0.067	1.720
F:M ratio (HC group; per point)	0.016	0.027	0.580	0.562	−0.038	0.069
Ff vs. FF model							Diagnostic criteria (Reference group: Not reported)						
2010 ACR/EULAR RA criteria	0.867	0.968	0.900	0.370	−1.029	2.763
1987 ACR criteria	0.854	0.906	0.940	0.346	−0.922	2.631
F:M ratio (RA group; per point)	0.108	0.101	1.060	0.288	−0.091	0.307
F:M ratio (HC group; per point)	−0.003	0.007	−0.380	0.706	−0.016	0.011

ACR, American Academy of Rheumatology; F:M ratio, female-to-male ratio; HC, healthy control; RA, rheumatoid arthritis; SE, standard error; VDR, vitamin D receptor.
