# Supplementary material for: Vitamin D Receptor Polymorphisms and Rheumatoid Arthritis Risk: A Systematic Review and Meta-Analysis Evaluating the Moderating Effects of Ethnicity, Bone Erosion, and Classification Criteria
Source: Arch Rheumatol. 2026 Apr 27;41(3):168–87. doi: 10.5152/ArchRheumatol.2026.25079 (PMC13401113; doi:10.5152/ArchRheumatol.2026.25079)
Supplement: Supplementary Material [file supplementary_material.pdf]

Supplementary Table 1. The detailed search query employed in this systematic literature review

Database	No.	Search Query	Results
PubMed	#1	"rheumatoid arthritis"[tiab] OR "Arthritis, Rheumatoid"[Mesh]	172,447
	#2	"vitamin D receptor"[tiab] OR VDR[tiab]	11,678
	#3	polymorphism*[tiab] OR TaqI[tiab] OR FokI[tiab] OR BsmI[tiab] OR ApaI[tiab] OR rs7975232[tiab] OR rs1544410[tiab] OR rs731236[tiab] OR rs2228570[tiab]	314,998
	#4	#1 AND #2 AND #3	61
Scopus	#1	TITLE-ABS-KEY ("rheumatoid arthritis")	234,743
	#2	TITLE-ABS-KEY ("vitamin D receptor") OR TITLE-ABS-KEY (VDR)	17,778
	#3	TITLE-ABS-KEY (polymorphism*) OR TITLE-ABS-KEY (TaqI) OR TITLE-ABS-KEY (FokI) OR TITLE-ABS-KEY (BsmI) OR TITLE-ABS-KEY (ApaI) OR TITLE-ABS-KEY (rs7975232) OR TITLE-ABS-KEY (rs1544410) OR TITLE-ABS-KEY (rs731236) OR TITLE-ABS-KEY (rs2228570)	606,601
	#4	#1 AND #2 AND #3	132
Web of Science	#1	AB="rheumatoid arthritis"	93,830
	#2	AB="vitamin D receptor" OR AB=VDR	11,224
	#3	AB=polymorphism* OR AB=TaqI OR AB=FokI OR AB=BsmI OR AB=ApaI OR AB=rs7975232 OR AB=rs1544410 OR AB=rs731236 OR AB=rs2228570	317,399
	#4	#1 AND #2 AND #3	53
Cochrane Registry	#1	"rheumatoid arthritis"	18,945
	#2	"vitamin D receptor" OR VDR	606
	#3	polymorphism* OR TaqI OR FokI OR BsmI OR ApaI OR rs7975232 OR rs1544410 OR rs731236 OR rs2228570	10,863
	#4	#1 AND #2 AND #3	4
Clinicaltrials.gov	Condition/disease	rheumatoid arthritis	-
	Other terms	vitamin D receptor polymorphism	-
	Intervention/treatment	-	-
	Total	All terms combined	0
Google Scholar	With all of the words	rheumatoid arthritis	-
	With the exact phrase	Vitamin D receptor	-
	With at least one of the words	polymorphism* TaqI FokI BsmI ApaI rs7975232 rs1544410 rs731236 rs2228570	-
	Total	-	200

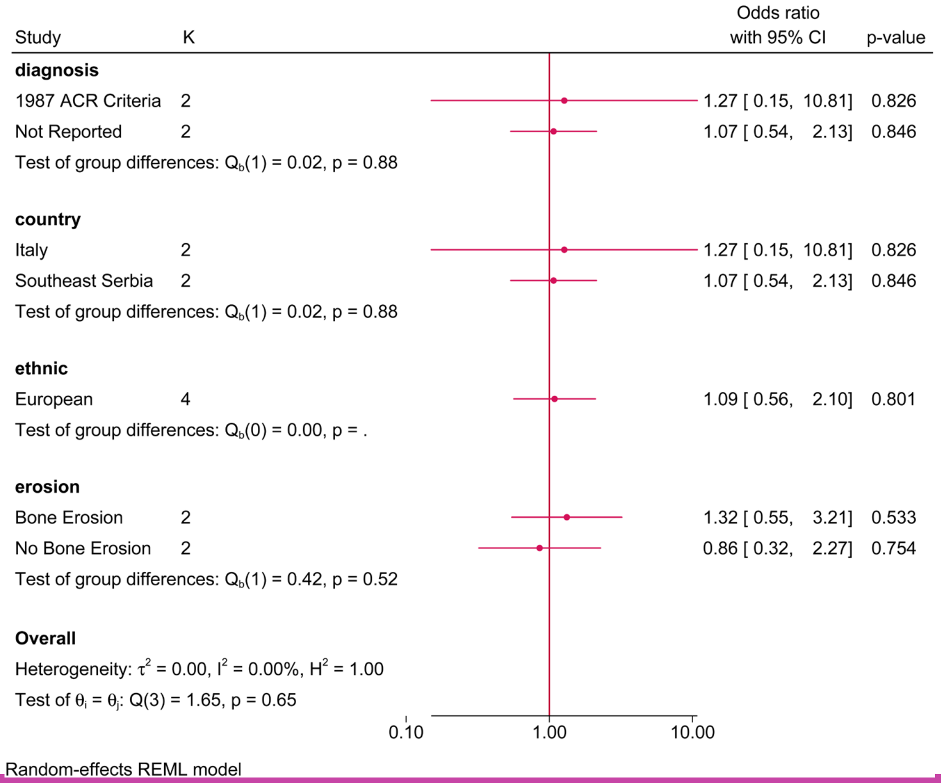

Supplementary Figure 1. Forest plot showing the association between the VDR gene – Apal – (aa vs. AA model) between rheumatoid arthritis patients and healthy control, stratified by classification criteria, country, ethnicity, and bone erosion.

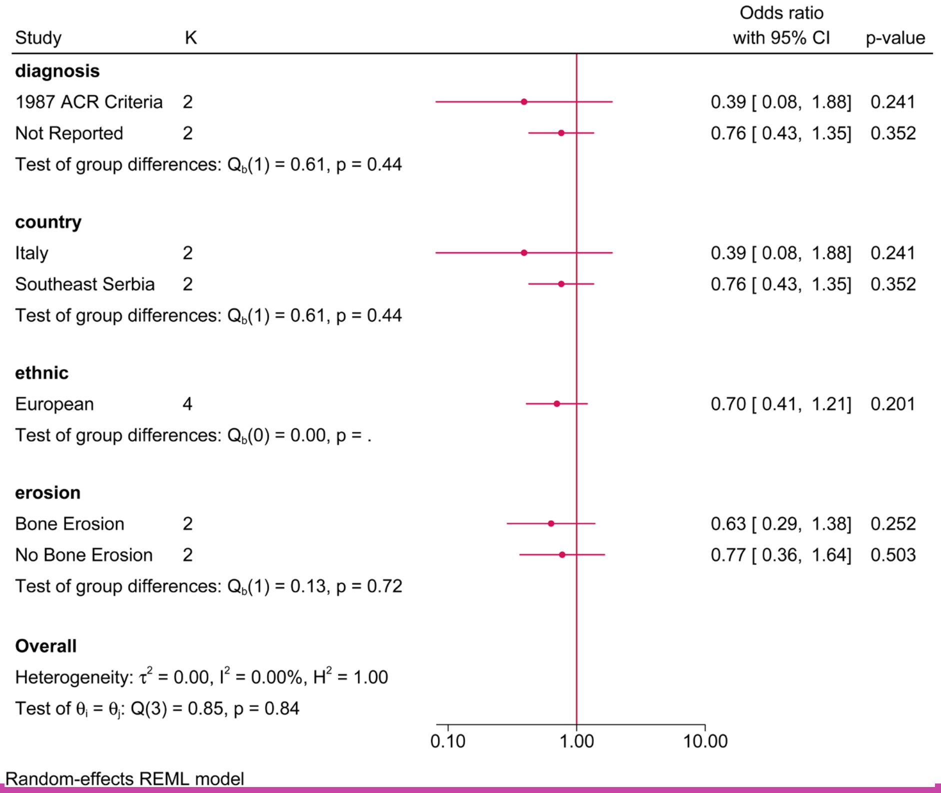

Supplementary Figure 2. Forest plot showing the association between the VDR gene – Apal – (Aa vs. AA model) between rheumatoid arthritis patients and healthy control, stratified by classification criteria, country, ethnicity, and bone erosion.

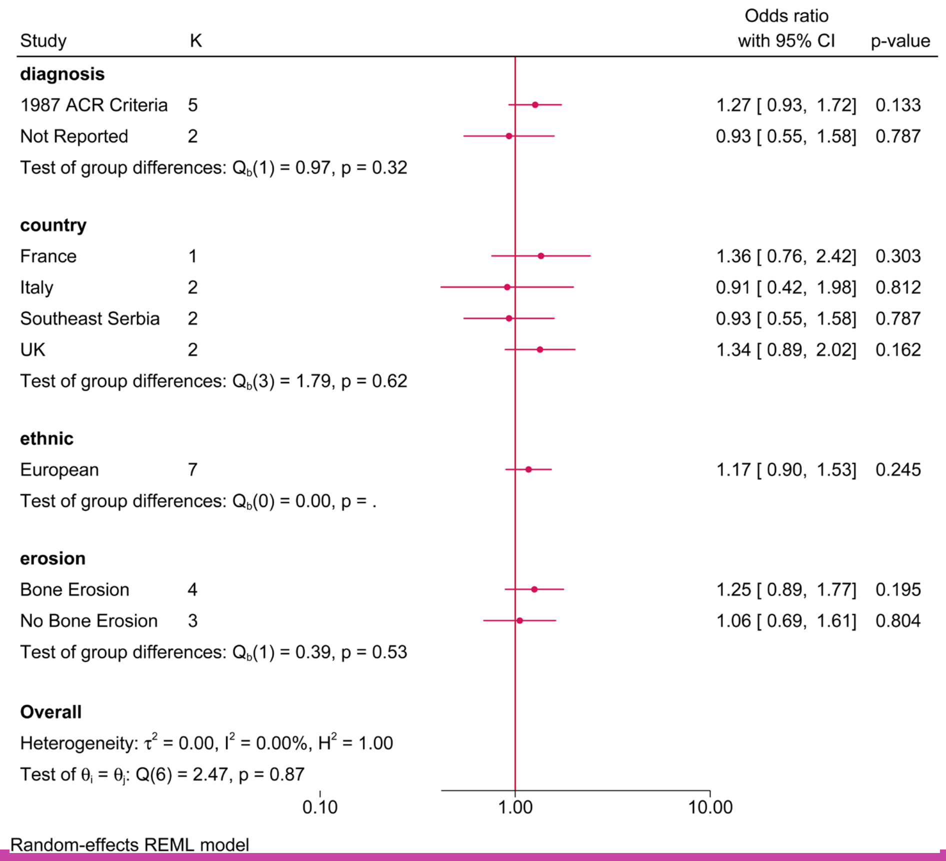

Supplementary Figure 3. Forest plot showing the association between the VDR gene – BsmI – (dominant model) between rheumatoid arthritis patients and healthy control, stratified by classification criteria, country, ethnicity, and bone erosion.

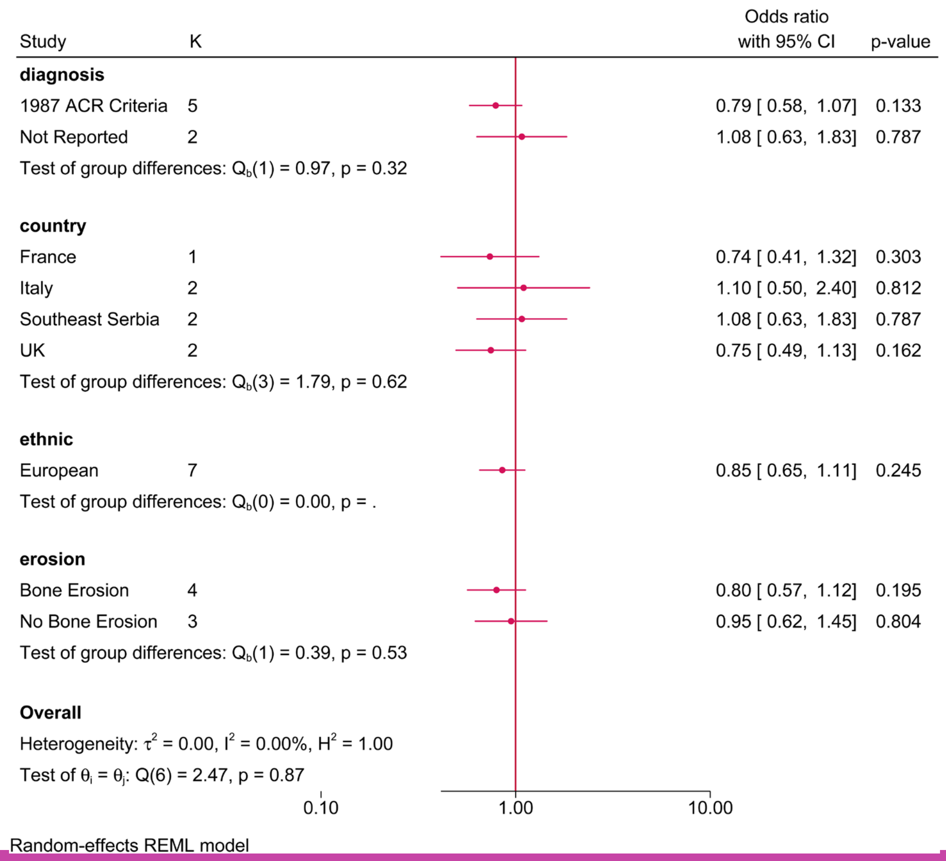

Supplementary Figure 4. Forest plot showing the association between the VDR gene – BsmI – (recessive model) between rheumatoid arthritis patients and healthy control, stratified by classification criteria, country, ethnicity, and bone erosion.

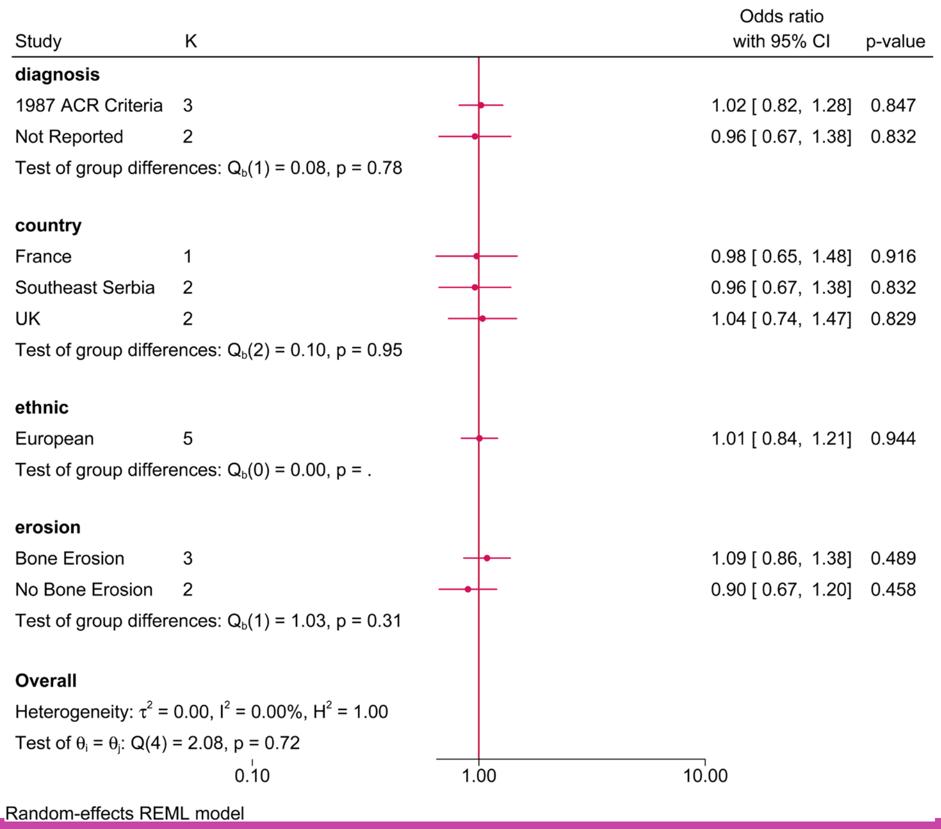

Supplementary Figure 5. Forest plot showing the association between the VDR gene – BsmI – (allelic model) between rheumatoid arthritis patients and healthy control, stratified by classification criteria, country, ethnicity, and bone erosion.

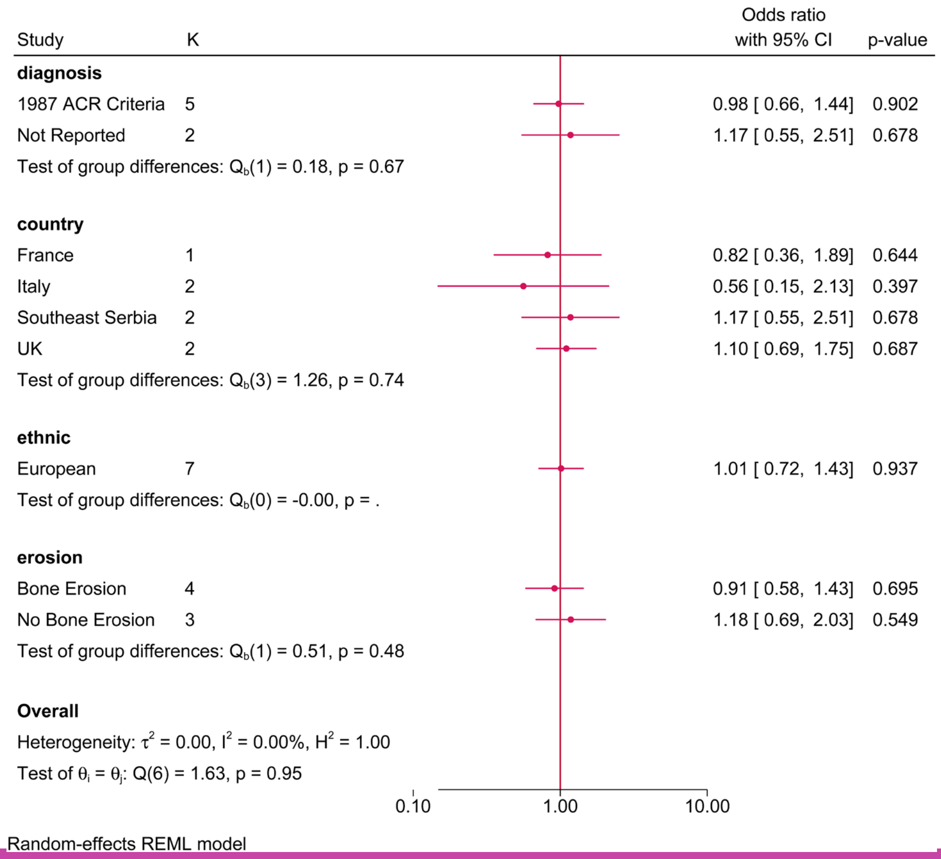

Supplementary Figure 6. Forest plot showing the association between the VDR gene – BsmI – (bb vs. BB model) between rheumatoid arthritis patients and healthy control, stratified by classification criteria, country, ethnicity, and bone erosion.

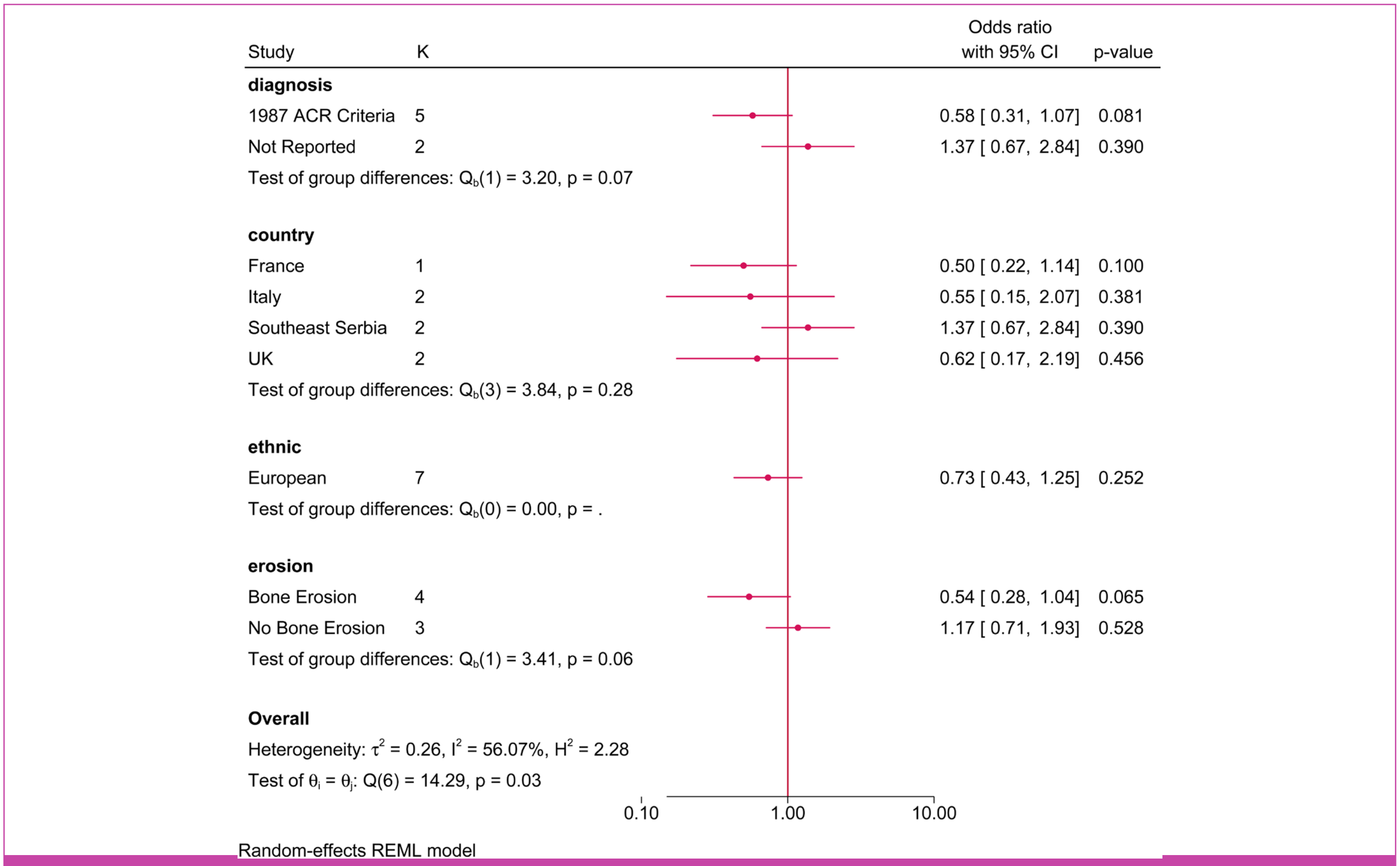

Supplementary Figure 7. Forest plot showing the association between the VDR gene – BsmI – (Bb vs. BB model) between rheumatoid arthritis patients and healthy control, stratified by classification criteria, country, ethnicity, and bone erosion.

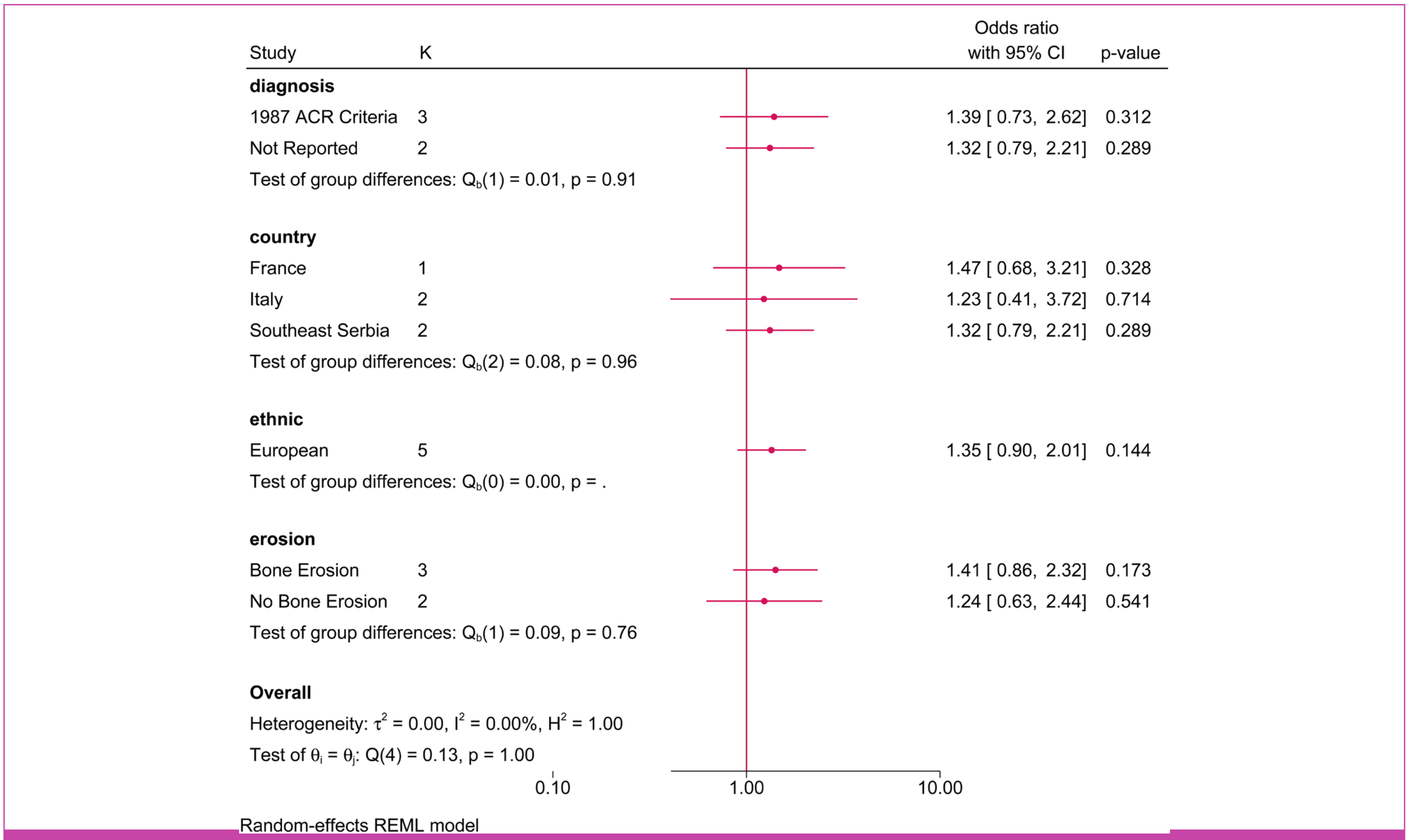

Supplementary Figure 8. Forest plot showing the association between the VDR gene – TaqI – (dominant model) between rheumatoid arthritis patients and healthy control, stratified by classification criteria, country, ethnicity, and bone erosion.

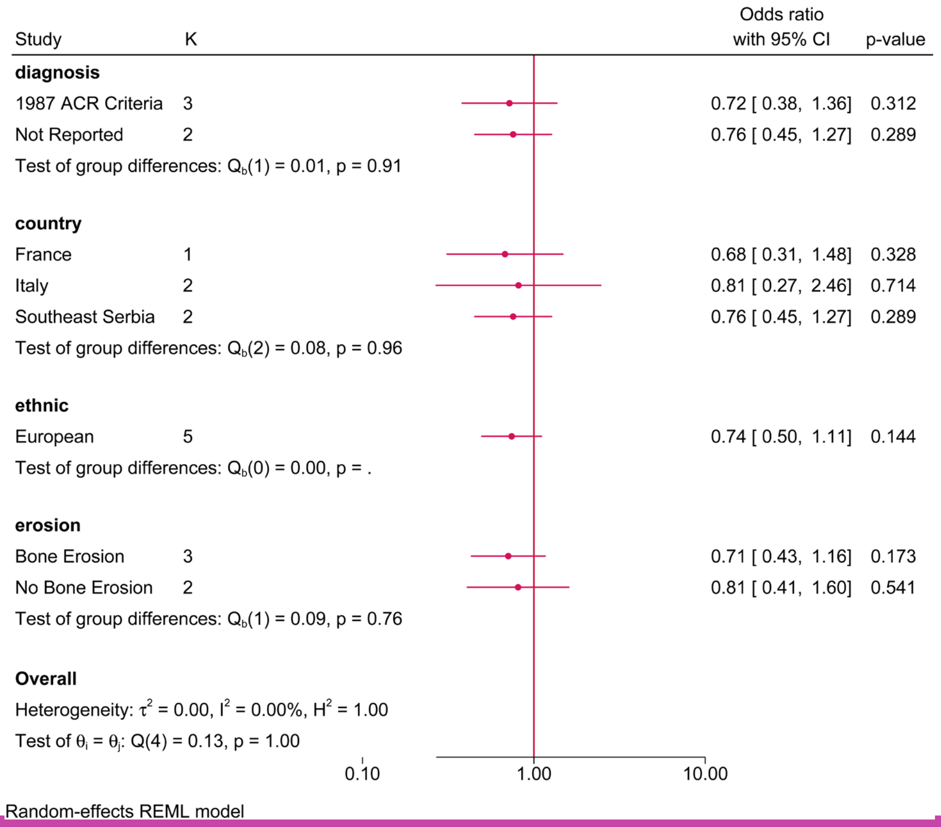

Supplementary Figure 9. Forest plot showing the association between the VDR gene – TaqI – (recessive model) between rheumatoid arthritis patients and healthy control, stratified by classification criteria, country, ethnicity, and bone erosion.

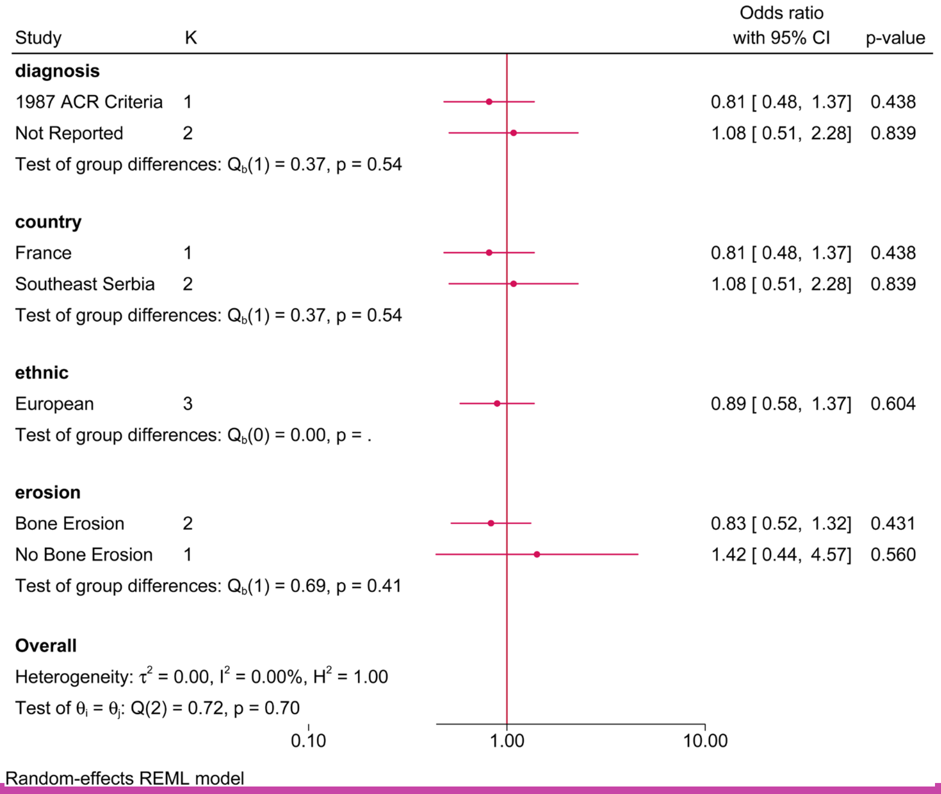

Supplementary Figure 10. Forest plot showing the association between the VDR gene – TaqI – (allelic model) between rheumatoid arthritis patients and healthy control, stratified by classification criteria, country, ethnicity, and bone erosion.

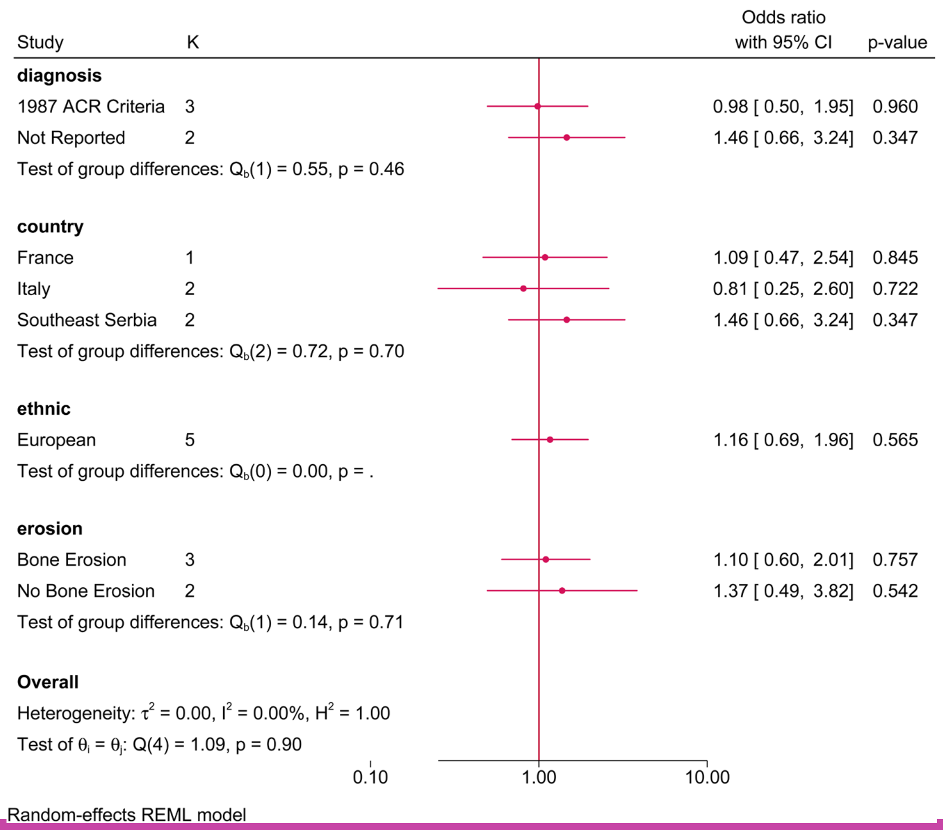

Supplementary Figure 11. Forest plot showing the association between the VDR gene – TaqI – (tt vs. TT model) between rheumatoid arthritis patients and healthy control, stratified by classification criteria, country, ethnicity, and bone erosion.
